# Metabolic profiling of zebrafish (*Danio rerio*) embryos by NMR spectroscopy reveals multifaceted toxicity of β-methylamino-L-alanine (BMAA)

**DOI:** 10.1038/s41598-017-17409-8

**Published:** 2017-12-11

**Authors:** Upasana Roy, Laura Conklin, Jürgen Schiller, Jörg Matysik, John P. Berry, A. Alia

**Affiliations:** 1 Institute for Medical Physics and Biophysics, University of Leipzig, D-04107 Leipzig, Germany; 2 Department of Chemistry and Biochemistry, Florida International University, North Miami, FL 33181 USA; 3 Institute of Analytical Chemistry, University of Leipzig, D-04103 Leipzig, Germany; 4 Leiden Institute of Chemistry, 2333 Leiden, The Netherlands

## Abstract

β-methylamino-L-alanine (BMAA) has been linked to several interrelated neurodegenerative diseases. Despite considerable research, specific contributions of BMAA toxicity to neurodegenerative diseases remain to be fully resolved. In the present study, we utilized state-of-the-art high-resolution magic-angle spinning nuclear magnetic resonance (HRMAS NMR), applied to intact zebrafish (*Danio rerio*) embryos, as a model of vertebrate development, to elucidate changes in metabolic profiles associated with BMAA exposure. Complemented by several alternative analytical approaches (i.e., *in vivo* visualization and *in vitro* assay), HRMAS NMR identified robust and dose-dependent effect of BMAA on several relevant metabolic pathways suggesting a multifaceted toxicity of BMAA including: (1) localized production of reactive oxygen species (ROS), in the developing brain, consistent with excitotoxicity; (2) decreased protective capacity against excitotoxicity and oxidative stress including reduced taurine and glutathione; (3) inhibition of several developmentally stereotypical energetic and metabolic transitions, i.e., metabolic reprogramming; and (4) inhibition of lipid biosynthetic pathways. Matrix-assisted laser desorption time-of-flight (MALDI-ToF) mass spectrometry further identified specific effects on phospholipids linked to both neural development and neurodegeneration. Taken together, a unified model of the neurodevelopmental toxicity of BMAA in the zebrafish embryo is presented in relation to the potential contribution of BMAA to neurodegenerative disease.

## Introduction

The non-proteinogenic amino acid, β-methylamino-L-alanine (BMAA), has received considerable attention based on reported linkages to several interrelated neurodegenerative diseases (ND). A possible role of BMAA in neurodegeneration was first proposed in a decades-long investigation of the high incidence of Amyotrophic Laterial Sclerosis (ALS) among Chamorro populations in Guam^1^. A contribution of BMAA to ALS, in this case, was initially based on the presence of this toxic amino acid in a cycad species (*Cycas micronesica*), and specifically a type of flour produced from the plant, and consumed widely among the Chamorro. In studies that followed, however, biomagnification of BMAA by frugivorous bats (*Pteropus mariannus*) - as consumers of the cycad’s fruit and, in turn, a delicacy in the region - was suggested as the route of exposure. And endosymbiotic cyanobacteria found in the cycad were, furthermore, identified as the biosynthetic source of the compound. Subsequent studies have similarly shown possible links between BMAA and various other cases of increased ND (e.g., Alzheimer’s Disease, Parkinson’s-like dementia)^2^. Moreover, studies indicating widespread production of BMAA by cyanobacteria^3^, and consequent biomagnification in diverse food webs, as a proposed vector for human exposure^1,4,5^, underscore the potentially important contribution of this toxic metabolite to these neurological disorders.

Although numerous studies have supported the proposed link between BMAA and ND, the toxicity of the compound, in relation to these diseases, remains to be clarified. The recognized neuroexcitatory activity of BMAA, and specifically agonistic interaction with glutamate receptors (GluR), has been largely suggested as a toxicological mechanism^6^. Levels of glutamate, as the endogenous ligand for GluR, are regulated at the synapse between neurons (via uptake and recycling by glial cells), but BMAA is not. Consequently sustained agonistic interaction of BMAA with GluR leads to post-synaptic excitotoxicity via influx of Ca^2+^, and subsequent oxidative stress resulting from production of reactive oxygen species (ROS) by mitochondria. More recently, several lines of evidence have suggested that aberrant incorporation of BMAA into proteins might represent an alternative mechanism for toxicity, as well as a possible means of bioaccumulation^5,7^. Specifically, it has been suggested that ribosomal misincorporation of BMAA into proteins may lead to accumulation of protein aggregates (e.g., fibrils, amyloid plaques) which are a hallmark - and putative etiological agent - of the various neurodegenerative diseases. This misincorporation has been shown most recently by radioactive labeling studies *in vivo* ^8^, and previously in cellular systems^7^, as well as *in vitro* protein synthesis experiments^9^. Several additional targets have been identified, and proposed to contribute to the link between BMAA and neurodegenerative disease. Most notably perhaps, antagonism of the cystine/glutamate antiporter $({{\rm{X}}}_{{\rm{c}}}^{-})$ was recently reported^10^. Inhibition of ${{\rm{X}}}_{{\rm{c}}}^{-}$ was, in this study, linked to both reduced uptake of cysteine (Cys) for biosynthesis of glutathione (by glial cells), as key protective mechanism against oxidative stress in neurons, and simultaneous accumulation of glutamate in the synapsis, which would consequently exacerbate GluR-mediated excitotoxicity.

In order to further elucidate the toxicity of BMAA, we utilized the zebrafish (*Danio rerio*) embryo, as a toxicological model, specifically coupled to nuclear magnetic resonance (NMR)-based metabolomics techniques, and more specifically high-resolution magic-angle spinning (HRMAS) NMR, as a novel means to identify metabolic changes associated with exposure to the toxin. The zebrafish model, in fact, has been previously used to demonstrate developmental effects of BMAA^11–13^, and initial studies demonstrated a range of morphological deformities, and other developmental (e.g., neurobehavioral) dysfunctions, among exposed embryos. Very recently, exposure to BMAA during early stages of zebrafish development was found to produce neurodevelopmental effects in both embryos, and later stages, including reduced neuronal outgrowth and subsequent behavioral effects at adult stages^14^. In addition, zebrafish embryos were recently employed in the proteomics studies of BMAA exposure, and BMAA was specifically found to affect proteins associated with GluR, oxidative stress and protein homeostasis^13^. In the present study, the application of ^1^H HRMAS NMR to the zebrafish embryo model represents the first non-invasive metabolomics study of BMAA. The NMR observations were further confirmed by *in vivo* and *in vitro* biochemical assays. Based on the data, a unified model of the neurodevelopmental toxicity of BMAA is proposed.

## Results

### HRMAS NMR Metabolic Profiling of BMAA-Exposed Zebrafish

Effects of BMAA on the metabolic profile of zebrafish were assessed at two relevant timepoints representative of early (i.e., *pharyngula* stage, ~27 hours post-fertilization [hpf]) and later eleutheroembryo/larval (i.e., 96 hpf) stages of embryo development. With respect to the CNS, in particular, the midbrain-hindbrain boundary are formed in the embryo by 27 hpf, and by 96 hpf elaboration of the CNS development is achieved including formation of telencephalon, mesencephalon, hypothalamus and, importantly, formation of primary and secondary motor neurons^15^. To evaluate dose dependency, two concentration levels of BMAA (BMAA_85_ and BMAA_170_, i.e., 85 and 170 µM) were evaluated. In the literature, a wide range of BMAA concentration for zebrafish embryo exposure have been mentioned^11–13^, and as a point of reference, Purdie *et al*. (2009) found that approximately 30% of zebrafish embryos were morphologically affected with 5000 μg/L (42 µM) BMAA^12^. Slightly higher (i.e., 2- and 4-fold) concentrations of BMAA were, therefore, choosen in the present study. At these concentration levels, however, no developmental toxicity in terms of morphological abnormalities, compared to controls, were observed for either developmental stage (Supplementary Fig. S1).

**Figure 1:**
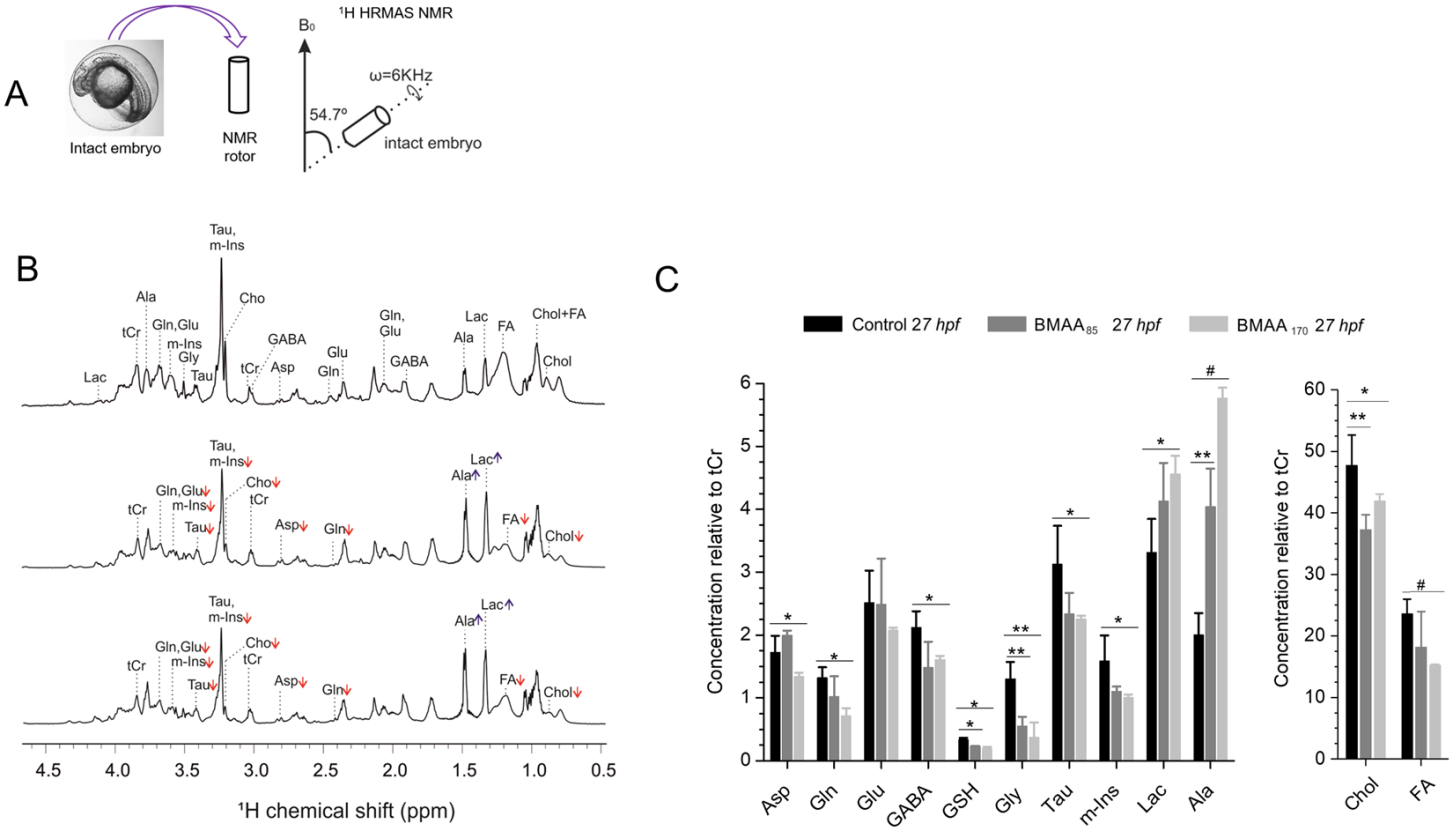
Effect of BMAA treatment on the metabolic profile of intact zebrafish embryos (27hpf). **(A)** Intact embryos with or without BMAA treatment were loaded into rotor and directly measured by HR MAS NMR at 600 MHz at a spinning speed of 6 kHz., cycle delay of 2 sec and total number of scan 256; **(B)** Representative ¹H HRMAS NMR spectra obtained after treatment of zebrafish embryos (3 h post fertilization) for 24 hours with no BMAA (control) (upper spectra), BMAA_85_ (middle) or BMAA_170_ (bottom). ^1^H shift was calibrated using TSP as an internal standard. Abbreviations: lactate (Lac), alanine (Ala), glutamine (Gln), glutamate (Glu), glycine (Gly), taurine (Tau), myo-inositol (m-Ins), choline (Cho), total creatine (tCr), gamma-aminobutyric acid (GABA), aspartate (Asp), glutathione (GSH), threonine (Thr), cholestrol (Chol), and fatty acid (FA). (**C**) The concentration of metabolites in control, BMAA_85_ and BMAA_170_ treated embryos. Statistical analysis (t-tests and ANOVAs) of the NMR quantification results were performed with OriginPro v. 8 (Northampton, USA). Value are average ± SE of mean (n = 9). (^#^ *P* < 0.001, **P < 0.01, *P < 0.05).

Metabolic profiles of intact embryos (3 hours post fertilization, hpf) treated with both BMAA concentrations for 24 h were evaluated by 1D and 2D ^1^H HRMAS NMR (Fig. 1A,B and Supplementary Fig. S2). Despite a lack of clear developmental toxicity (e.g., morphological abnormalities; Supplementary Fig. S1), 1D ^1^H HRMAS-NMR of BMAA-treated embryos at 27 hpf identified several robust changes in metabolic profiles compared to controls (Fig. 1B). Two-dimensional homonuclear correlation spectroscopy (¹H-¹H COSY) enabled confirmation of metabolites, and improved resolution of metabolite profiles, based on ^1^H-^1^H cross peaks which overcome extensive second order coupling that, otherwise, complicates the 1D spectrum (Supplementary Fig. S2). Accordingly, 2D ^1^H-^1^H COSY confirmed changes observed in the 1D spectra with BMAA treatment (Supplementary Fig. S3–5). Subsequent quantification of the most abundant metabolites are shown in Fig. 1C. Significant decreases in the levels of aspartate (Asp), glutamine (Gln), gamma-aminobutyric acid (GABA), glutathione (GSH), glycine (Gly) taurine (Tau), myo-inositol (m-Ins), cholesterol (Chol) and free fatty acids (FA), and increased levels of lactate (Lac) and alanine (Ala), at 27 hpf were observed in the BMAA treated embryo. Although a dose-dependent effect was suggested for most metabolites, significant differences were generally only observed between highest exposure concentration (i.e., 170 μM BMAA) and controls with the exception of observed decreases in GSH and Gly which were statistically significant at both exposure concentrations, and observed dose-dependent increase in Ala (Fig. 1C).

**Figure 2:**
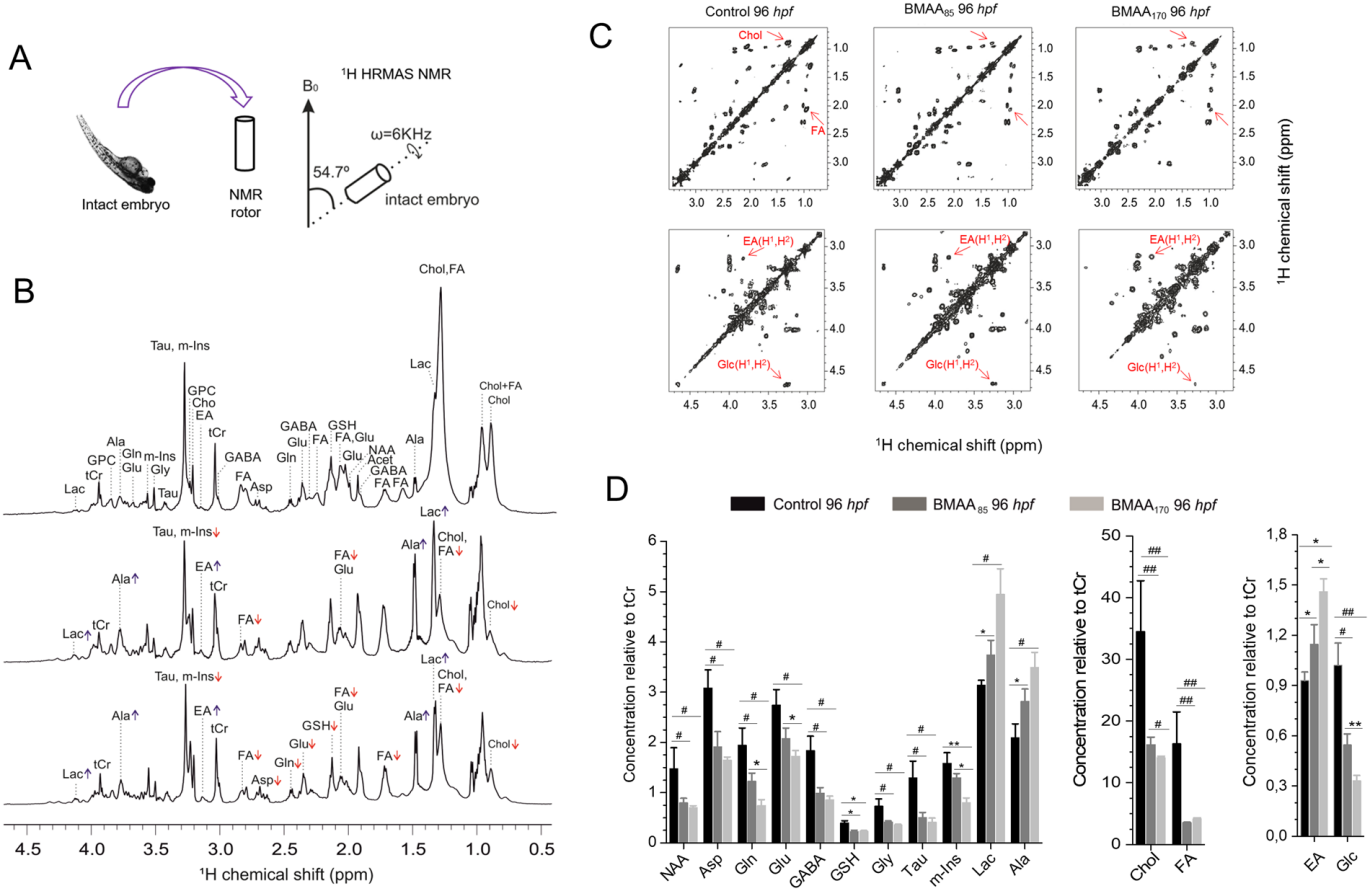
Effect of BMAA treatment on the metabolic profile of intact zebrafish embryos (96hpf). **(A)** Intact embryos with or without BMAA treatment were loaded into rotor and directly measured by HR MAS NMR at 600 MHz at a spinning speed of 6 kHz. cycle delay of 2 sec and total number of scan 256; **(B)** Representative ¹H HRMAS NMR spectra obtained after treatment of zebrafish embryos (72 h post fertilization) for 24 hours with no BMAA (control) (upper spectra), BMAA_85_ (middle) or BMAA_170_ (lower). ^1^H shift was calibrated using TSP as an internal standard. Reduction of essential amino acids (Asp, Gln, GSH, Tau, m-Ins, Gly, Glu and GABA) and lipids (Chol and FA) and increase in Lac, Ala and EA were prominent in BMAA treated embryo compared to controls. (**C**) 2D ^1^H-^1^H COSY spectra showing clear reduction of Chol and FA and glucose (Glc) and an increase in EA in BMAA treated embryos. (**D**) The concentration of metabolites in control, BMAA_85_ and BMAA_170_ treated embryos measured from HR MAS NMR spectra. Statistical analysis (t-tests and ANOVAs) of the NMR quantification results were performed with OriginPro v. 8 (Northampton, USA). Value are average ± SE of mean (n = 9). (^##^ *P* < 0.0001, ^#^ *P* < 0.001, **P < 0.01, *P < 0.05).

In order to evaluate possible influence of developmental stage, metabolic profiles of eleuthero-embryos (72 hpf) treated with BMAA_85_ and BMAA_170_ for 24 h (96 hpf total) were concurrently evaluated (Fig. 2A,B). Alongside 1D NMR, ^1^H-^1^H COSY and *J-*resolved spectra were recorded, and enabled improved spectral dispersion and confidence in metabolic identification and quantification (Supplementary Figs S6–8; Supplementary Table S1).

Despite differences in metabolic profile between the two stages (Supplementary Fig. S9), the same relative changes (i.e., decreases and increases) in metabolites following BMAA exposure, compared to controls, as observed at 27 hpf were identified in 96 hpf embryos (Figs 1 C and 2D). A significant increase in ethanolamine (EA), and decreases in both N-acetylaspartate (NAA) and glucose (Glc), were additionally identified at 96 hpf (Fig. 2D). Resolution of EA and Glc was specifically enabled by 2D ^1^H-^1^H COSY (Fig. 2C), and not discernible in 1D spectra. Similarly, the detection of dose-dependent reductions in FA and Chol, and increases in Lac and Ala, were prominently reinforced by 2D spectra (Fig. 2C, and Supplementary Fig. S10).

A highly reproducible pattern of changes in metabolic profiles, relative to controls, at both developmental stages underscores the robustness of the ^1^H HRMAS NMR metabolomics approach. Comparison of the metabolic profiles between the two developmental stages, however, suggests that dose dependency of the effects of BMAA was greatly enhanced for 96 hpf embryos. As evidence of this, significant changes between not only controls and BMAA_170_, but also controls and BMAA_85_, and between the two exposure concentrations, were observed; and higher levels of statistical significance (p < 0.0001) were observed in most cases. Details of ANOVA results at the two stages are shown in Supplementary Table S2. Simply stated, a more pronounced effect of BMAA is observed at the 96 hpf stage, and subsequent studies (i.e., assessments of oxidative stress and lipid profiles; see below) focused, therefore, on this later developmental stage.

To probe if control and BMAA-treated embryos can be discriminated (for group separation), and to determine the spectral regions (and corresponding compounds) mainly responsible for the separation, the HRMAS spectra at both stages (27 and 96 hpf) were investigated by multivariate analysis with PLS-DA modeling. The PLS-DA score plot of the of first two principle components explains 57% of the total variance, and a clear clustering of the control and treated embryo groups could be observed in the score plot of PLS-DA1 vs PLS-DA2. The details of this analysis are provided in Supporting Information (Supplementary Figs S11 and S12).

### Oxidative Stress in BMAA-Exposed zebrafish Embryos

**Figure 3:**
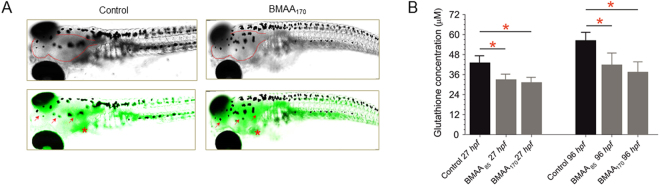
Localization of reactive oxygen species (ROS) production in zebrafish embryos exposed to BMAA_170_ as compared to control embryos. (**A**) Embryos were incubated for 60 min in CM-H2DCFA (10 μM) in rearing medium. Control embryo (left), i.e., water only, bright field image (top) and overlay with fluorescence (bottom). BMAA_170_-treated embryo (right), bright field image (top) and overlay with fluorescence (bottom). Red outline highlights the brain region. Red arrow indicates fluorescent cells in the brain region surrounding the pigments. Asterisk indicate fluorescent cells in the yolk sac. (**B**) Intact embryos were homogenized to separate metabolic layer in 5% 5-Sulfosalicylic Acid (SSA) solution and glutathione (GSH) levels were analysed by using GSH assay kit from Sigma-Aldrich. Significant reduction of GSH (p < 0.001; n = 9) in BMAA treated embryo is clearly observed.

Oxidative stress as a mechanism for BMAA toxicity was evaluated based on the production ROS, and modulation of glutathione (as a key cellular protective mechanism against oxidative stress). Generation of ROS was assessed *in vivo* using a previously developed method based on the fluorescent probe, chloromethyl-2′,7′-dihydrodichlorofluorescein diacetate (CM-H_2_DCFDA), adapted to the zebrafish embryo model^16^. As shown in Fig. 3A, increased levels of ROS were observed in embryos treated with BMAA_170_, compared to controls, and specifically within the region of the developing brain. This observation is consistent with both specific uptake and targeting of BMAA to the brain (and reported ability to readily cross the blood-brain barrier), and consequent Ca^2+^ induced production of ROS by mitochondria associated with GluR excitotoxicity. Since increase of ROS may be exacerbated by reduction of GSH, a total glutathione measurement was performed by way of a well-established *in vitro* colorimetric assay. As shown in Fig. 3B, exposure to BMAA_170_ significantly reduced total glutathione levels compared to controls (p < 0.001). This decline is consistent with, and generally confirms, the observed decrease in GSH measured by HRMAS NMR in BMAA treated embryos (Fig. 2).

### Lipid Profiles of BMAA-Exposed Zebrafish Embryos

Based on the observed decreases in FA and Chol by HRMAS NMR, as well as biosynthetic precursors (i.e., NAA, m-Ins, EA) of lipid metabolism, lipid profiles in BMAA-exposed zebrafish embryos were further characterized. Lipid characterization methods targeted 96-hpf embryos since, at this stage, not only is lipid (i.e., FA, Chol) depletion dose dependent (Fig. 2D), but NAA, as a primary precursor of brain lipid synthesis, is significantly reduced.

**Figure 4:**
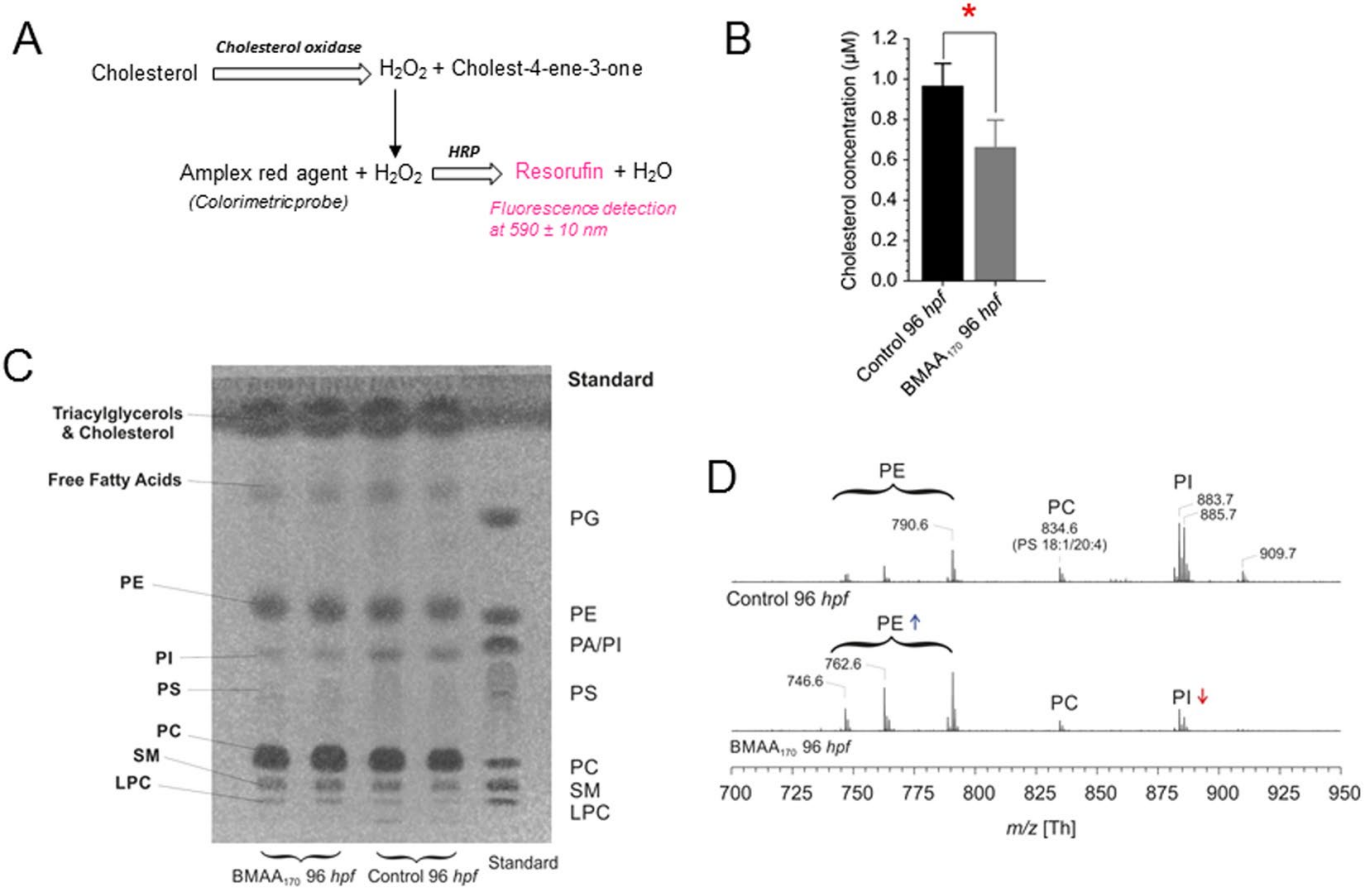
Analysis of lipid profile with BMAA_170_ treated embryo. Intact 96 hpf embryos were homogenized to separate lipid and are distributed in two parts. One part is dried and used for HPTLC and MALDI-ToF analysis of lipids and other part is suspended in 100 mM phosphate buffer and distributed in 96 well plates for cholesterol assay. **(A)** Reaction diagram of the enzyme activity assay of cholesterol measured photometrically at 590 ± 10 nm. **(B)** Quantification of cholesterol using Amplex red agent based enzymatic assay lit. Significant reduction of cholesterol (p < 0.001; n = 9) in BMAA_170_ treated embryo is observed. **(C)** Image of a HPTLC plate of a reference PL mixture (1.69 mg each) and the embryo lipid extracts. **(D)** Comparison of negative ion MALDI-ToF lipid profiles in BMAA_170_-exposed zebrafish embryos, relative to controls. Phosphatidylinositol (PI) lipids are decreased and phosphatidylethanolamine (PE) lipids are increased in BMAA_170_ treated embryos compared to controls.

Total cholesterol was measured by a commercially available colorimetric assay using Amplex® Red (Fig. 4A), and results show a significantly decreased (by more than 25%, p < 0.001) total cholesterol pool (Fig. 4B). This result aligns with the observed decrease of Chol in the NMR experiments (Fig. 2).

Lipid profiles were further characterized by matrix-assisted laser desorption ionization/time-of-flight (MALDI-ToF) mass spectrometric analysis, and MALDI-ToF coupled to high-performance thin layer chromatography (HPTLC) as previously described^17,18^. This approach was previously utilized by our group to characterize lipid profiles (alongside NMR) in the brain of adult zebrafish, and identified high degree of similarity with humans, concluding zebrafish was a good model for brain lipid chemistry^19^. Chromatograms of a reference mixture of several known phospholipids (PLs), and lipid extracts from control versus BMAA_170_-treated zebrafish embryos (at 96 hpf), are shown in Fig. 4C. Good chromatographic separation of the individual PL is evident, and phosphatidylcholines (PC) were found to be the most prominent phospholipids. The positive- and negative-ion MALDI-ToF mass spectra of selected brain lipid fractions (obtained directly from the developed TLC plate) are shown for BMAA_170_ treated embryo lipid extracts in Supplementary Fig. S13, and the assignments of all detected major peaks are provided in Supplementary Table S3. Information about the fatty acyl residues within a lipid can be easily obtained by negative-ion MALDI-ToF mass spectrometry, although not all lipid species can be easily detected as negative ions. For this reason, only negative-ion MALDI-ToF mass spectra of phospatidylethanolamine (PE) and phosphatidylinositol (PI) lipids are shown (Supplementary Fig. S13), and the observed peaks directly assigned to the corresponding lipid species.

Comparison of HPTLC/MALDI-ToF lipid profiles in BMAA-exposed zebrafish embryos reveals a notable observation with respect to the level of PI and PE. As shown in Fig. 4D, the level of PI is decreased, while the level of PE clearly increased, in BMAA-exposed embryos (Fig. 4D). The quantitative decrease in PI, and increase in PE, is noteworthy in light of simultaneous decrease in m-Ins and increase in EA (measured by HRMAS NMR; Fig. 2) as biosynthetic building blocks, respectively, of these groups of PL.

## Discussion

**Figure 5:**
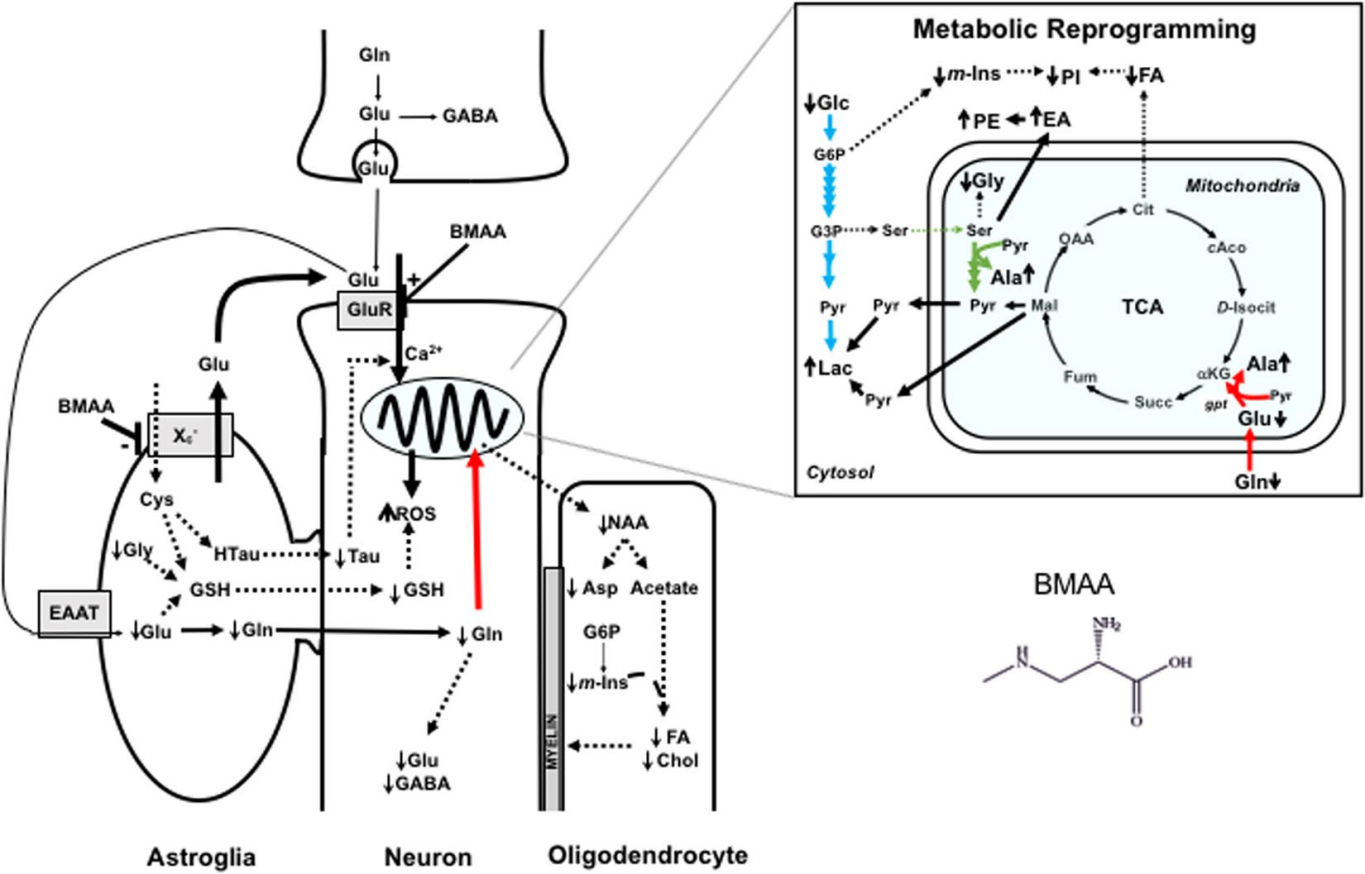
Proposed model of the multifaceted toxicity of BMAA in the central nervous system as evidenced by observed changes in metabolic profile in the zebrafish embryo model, and supported by previous studies. As previously shown^6^ BMAA disrupts Ca^2+^ homeostasis in neurons via agonistic interactions with GluR, causing mitochondrial dysfunction including release of ROS. Increase in ROS was prominent in the brain region of BMAA_170_ treated embryo (Fig. 3A) which is consistent with excitotoxic mitochondrial dysfunction. This excitotoxicity subsequently interferes with stereotypical metabolic transitions (i.e., metabolic reprogramming) associated with neuronal differentiation^27^ as suggested in the current study by increased glycolysis (i.e., decrease in glucose, and increase in lactate), glutaminolysis (i.e., decrease in Glu and Gln, and increase in Ala and Gln/Glu ratio) and serinolysis (i.e., increase in Ala, and decrease in Gly) in BMAA-treated embryos (Fig. 2D). It has been previously shown that BMAA inhibits the Cyst/Glu antiporter (X_c_¯) of astroglia which limits supply of Cys for biosynthesis of both GSH and Tau to the neuron^10^, and consequently exacerbates oxidative stress (i.e., ROS production) and disruption of Ca^2+^ homeostasis, respectively. Glutathione depletion is observed in the present study (Figs 2D and 3B), and likely compounded by reduced levels (Fig. 2D) of Gly (via serinolysis) and Glu (via glutaminolysis) as biosynthetic precursors of GSH. Reduction of Gln would, in addition, lead to reduction of the essential neurotransmitters, Glu and GABA. Mitochondrial dysfunction additionally reduces production of NAA (Fig. 2D) which is the biosynthetic precursor of lipid metabolism. Consistent with reduced NAA, both FA and Chol were significantly decreased (Figs 2D and 4B). NAA is produced in the mitochondria of neurons, and transported to oligodendrocytes where it is metabolized into Asp and acetate; acetate is utilized for the synthesis of FA and Chol which, in turn, are utilized for myelin synthesis, as well as essential components of cell membranes, and key regulatory molecules. Among the lipids characterized by HPTCL/MALDI-ToF (Fig. 4C–D), phospholipids levels were affected by BMAA: PE increased, and PI decreased. The former may be linked to a compensatory increase in the synthesis of EA, and the latter to metabolic diversion of the m-Ins precursor (G6P) by elevated glycolysis. Observed increases and decreases in metabolites shown by arrows (i.e., ↑ and ↓, respectively). Pathways elevated by BMAA, according to the proposed model indicated by heavy lines, and those attenuated by dashed lines. Pathways associated with metabolic reprogramming by BMAA colored as follows: blue = glycolysis, red = glutaminolysis and green = serinolysis. Abbreviations: glucose-6-phosphate (G6P); glyceraldehyde-3-phosphate (G3P); pyruvate (Pyr); malic acid (Mal); fumaric acid (Fum); succinic acid (Suc); α-ketoglutarate (αKg); D-isocitrate (D-Isocit); cis-aconitate (cAco); citric acid (Cit); oxaloacetic acid (OAA); glutamate pyruvate transaminase (GPT); glutamate receptors (Glu-R); excitatory amino acid receptor (EAAT); tricarboxylic acid cycle (TCA).

Converging lines of evidence point to a contribution of BMAA to neurodegenerative disease^1–3,5^. In this regard, two mechanisms of BMAA toxicity have been generally proposed: neuronal “excitotoxicity” via glutamatergic pathways^6,20^, and misincorporation of this unusual amino acid into proteins during synthesis/translation^5,7^. In the present study, we provide new mechanistic insight into BMAA neurotoxicity by exploring via state-of-the-art HRMAS NMR, and complementary *in vitro* and *in vivo* assays, the comprehensive metabolic network of intact zebrafish embryos. Taken together, the metabolomics approach enabled a unified model (Fig. 5) of the apparently multifaceted toxicity of BMAA directly relevant to neurochemical networks, and thus, possible role in neurodegeneration.

Despite a lack of apparent developmental toxicity (i.e., morphological abnormalities), 1D and 2D NMR clearly identified several robust, dose- and stage-dependent alterations in metabolite profiles of BMAA-exposed embryos. Interestingly, the lack of observable developmental toxicity in the present study (Supplementary Fig. S1) contrasts with the earliest studies of BMAA in the zebrafish model^11,12^; however, a similar lack of morphological abnormality in BMAA-exposed zebrafish has, in fact, been reported in more recent studies^13^. The ability of HRMAS NMR to discern and rigorously quantify major metabolites in the intact zebrafish embryo has been previously demonstrated, by our group, specifically with respect to the similarly cyanobacteria-derived polymethoxyalkenes as teratogenic metabolites^21^. The present study not only generally reinforces the robustness of HRMAS NMR in the metabolic profiling of intact zebrafish, but furthermore, demonstrates the capabilities of this technique to identify and quantify – even in the absence of otherwise clear, e.g., morphological, toxic effects - a broad range of toxicologically relevant endpoints inaccessible by other techniques.

Metabolic effects observed in the current study are, generally speaking, consistent with three interrelated networks including: (1) *metabolic reprogramming* associated, in particular, with stereotypical energetic transitions of development; (2) protective mechanisms associated with excitotoxicity and/or oxidative stress; and (3) biosynthetic pathways associated with relevant lipids. More generally, the observed metabolic changes, along with complementary studies, support excitotoxicity as a mechanism of toxicity. Consistent with excitotoxicity – and consequent oxidative stress - zebrafish embryos (at 96 hpf) exposed to BMAA were characterized, for example, by elevated ROS in the brain region specifically (Fig. 3A). Generation of ROS, accompanied by increased Ca^2+^ influx (i.e., agonism of GluR), following BMAA exposure has been previously demonstrated in several systems, and these studies generally point to excitotoxic mitochondrial disruption as a central link in this regard. And, in turn, mitochondrial dysfunction generally aligns with all NMR-observed changes in metabolic profiles of BMAA-exposed zebrafish. Prior studies^22^ have suggested that mechanisms other than GluR-mediated excitotoxicity, and specifically proteotoxicity as an alternative mechanism, may contribute (particularly at lower exposure concentrations) to the oxidative stress associated with BMAA exposure. The role of protein misincorporation (i.e., proteotoxicity) was not specifically investigated, or evidenced by any of the observed metabolic changes at the test concentrations used, in the current study. Although metabolic profiles were measured for whole embryos, several lines of evidence suggest that observed effects are particularly relevant to neural cells, and hence neurotoxicity. In support of this, for example, a number of the altered metabolites are found almost exclusively (e.g., NAA) or greatly enriched (e.g., Tau, GABA) in neurons^23^, and the observed increase of ROS in the brain, moreover, are a clear indication of altered neurochemistry with BMAA treatment (Fig. 3A).

### Metabolic Reprogramming by BMAA

One overarching pattern to emerge in HRMAS NMR studies was the alteration of numerous metabolites associated with so-called *metabolic reprogramming*. First identified by Warburg^24^ in 1956 in relation to tumor cells, “metabolic reprogramming” is characterized by a shift away from oxidative phosphorylation, and toward a reliance on aerobic glycolysis and related pathways, to supply the energetic and metabolic needs of cell proliferation^25^. And, indeed, NMR-based metabolomics approaches have been previously used to understand metabolic reprogramming, as it relates to cancer, in a range of systems. Although the so-called “Warburg Effect” is largely associated with cancer cell metabolism, several lines of evidence have confirmed a similar metabolic transition, and in effect, a reverse shift (from aerobic glycolysis to oxidative phosphorylation), during normal neural development^26–28^. Underscoring its essential importance during neural development, experimental inhibition of this metabolic transition triggers apoptotic cell-death in neural progenitor cells^28^.

Metabolite profiles observed in NMR studies suggest inhibition of this requisite metabolic transition by BMAA, and thereby, implicate this inhibition in BMAA neurotoxicity. Consistent with increased mitochondrial aerobic glycolysis, an increase in Lac with a concurrent decrease in Glc was observed for 96-hpf embryos treated with BMAA. Previous studies show that, in the brain, Lac produced via lactate dehydrogenase (LDH) fermentation of pyruvate, and not pyruvate itself, is the end product of aerobic glycolysis^29,30^. In the fully developed brain, Lac produced from Glc by aerobic glycolysis in glial cells (i.e., astrocytes, oligodendrocytes) – and supplied to neurons via the so-called “astrocyte-neuron lactate shuttle” - is the primary energy source for mitochondrial respiration in neurons, as well as an essential biosynthetic substrate^31–33^. It has been, likewise, shown that blood-supplied (as opposed to glial derived) lactate is a similarly key energy source during neural development, and the supply of Lac is, furthermore, essential to myelinogenesis in glial cells during embryo development^34^. Taken together with previous findings, the increase in Lac observed here suggests that BMAA leads to an increase in glycolysis to fulfill energy requirements to sustain cell functioning, specifically in response to presumptive mitochondrial disruption (*vis á vis* “excitotoxicity”).

Alongside glycolysis, it has been shown that metabolic programming - including that which occurs during neural differentiation - additionally co-opts *glutaminolysis* (i.e., mitochondrial Gln uptake, and subsequent metabolism via Glu to α-ketoglutarate for entry to the TCA cycle) to meet energy and metabolic demands^27,35,36^. Consistent with increased glutaminolysis^37^, our results show decrease in the total levels of Gln and Glu (Fig. 2) and an increase of Glu/Gln ratio (Supplementary Fig. S14), in BMAA-treated, 96-hpf embryos. More broadly, the observed decreases of not only Glu and Gln, but also GABA (Fig. 2), are likely linked by way of the so-called “glutamate/GABA-glutamine cycle”. It is well established that Gln is synthesized in astrocytes from Glu and ammonium (via glutamine synthetase) and, in turn, Gln leaves the astrocytes, and is transported into neurons where it is hydrolysed by glutaminase to release Glu and ammonium. In case of GABAergic neurons, the neurotransmitter, GABA, is subsequently synthesized from Glu (via glutamic acid decarboxylase [GAD]). Postsynaptically, Glu and GABA from synapses are taken-up by glia (mainly astrocytes), and finally converted back to Gln.

Catabolism of Glu and Gln by glutaminolysis would, furthermore, be expected (as is observed) to accompany increased consumption of Glc. In the brain, Glu is the most abundant and important excitatory neurotransmitter in the brain, whereas GABA (derived from Glu via Gln) is the most abundant inhibitory neurotransmitter. Accordingly, the two metabolites are essential mediators of neuronal activity in the adult brain, but are known to play, in addition, a key role in neural development. As such, under normal physiological condition, the majority of the Glc utilised (via aerobic glycolysis) in the CNS is coupled to the Glu–Gln cycle, and particularly the endergonic, ATP-dependent synthesis of Gln from Glu in glia^38^. It is, accordingly, proposed that depletion of Glu and Gln (via glutaminolysis) would, in turn, lead to further upregulation of glycolytic consumption of Glc (as observed) to maintain homeostasis of these key neurotransmitters during development.

Elevated levels of Ala (as observed by NMR) would, likewise, be consistent with increased glutaminolytic consumption of Glu and Gln. Alanine is the major transporter of ammonium in the brain including an established role in the Glu-Gln cycle^39^. As such, elevated biosynthesis of Ala might represent a compensatory mechanism in the CNS to supply ammonium to the glial cells to maintain Glu/Gln homeostasis. Alternatively, it is known that Glu is deaminated (to α-ketoglutarate) during glutaminolysis by three pathways, and utilization of one of these pathways - namely glutamate pyruvate transaminase (GPT) –utlizes transamination of pyruvate to Ala. Deamination of Glu by GPT would, thereby, further explain elevated levels of Ala. Notably, a similarly pronounced increase of Ala was, in fact, previously observed in experiments utilizing rat brain slices after incubation BMAA^40^.

Finally with respect to metabolic reprogramming, glycolysis and glutaminolysis are also known to be coupled to a third catabolic process, namely *serinolysis*. In serinolysis, serine (Ser) is catabolically utilized to produce pyruvate which subsequently enters (via glycerate 2-phosphate) the glycolytic pathway. Although Ser itself was not discernable by HRMAS NMR (in either treated or control embryos), alterations of other metabolites observed in the present study are consistent with a possible role of this pathway. In particular, a decrease in Gly which is directly biosynthesized from Ser aligns with metabolic depletions of Ser. Similar to Glu and GABA, the neurotransmitter Gly has, furthermore, been shown to have a key role in both CNS function and neural development^41^. In addition, Ala is produced (via transamination of pyruvate) during catabolism of Ser, and increased serinolysis could potentially explain, along with a shift to glutaminolysis, the significantly increased levels of Ala (Fig. 2). A role of serinolysis in the observed metabolic effects of BMAA is particularly compelling as serine has, in fact, been suggested to rescue neuronal cells from BMAA toxicity^42^, and even potentially “slow disease progression”,^43^ although this has largely been linked to a purported misincorporation of BMAA (in place of serine) into proteins. Moreover, compared to glycolysis and glutaminolysis, serinolytic pathways remain relatively unresolved, and any contributions of this pathway in the metabolic effects of BMAA, likewise, remain to be investigated in future studies.

### Attenutation of Protective Mechanisms Against Excitotoxicity

Aside from essential roles in CNS function and development, Glu and Gly are biosynthetic precursors of GSH. Observed decreases in the two may, therefore, explain the observed decrease in GSH in BMAA-treated embryos (Figs 1C, 2D and 3B). Moreover, GSH is key protective mechanism of neurons^44^ and the resultant depletion may consequently contribute to the excitotoxicity – and, in particular, oxidative stress, i.e., generation of ROS (Fig. 3A) – associated with BMAA. Glutatione is, in fact, biosynthesized (as a tripeptide) from Glu, Gly and Cys, and although Cys could not be discerned by HRMAS NMR (in treated or control embryos), previous studies have demonstrated inhibition of the ${{\rm{X}}}_{{\rm{c}}}^{-}$ antiporter which directs uptake of Cys (as the dimer, cystine) to astrocytes to supply biosynthesis of GSH. It is proposed, therefore, that concurrent inhibition of ${{\rm{X}}}_{{\rm{c}}}^{-}$ transport, and observed reductions in Glu and Gly, would contribute to GSH depletion, and further exacerbate oxidative stress, associated with BMAA.

Interestingly, alongside decreased GSH, a significant decrease in Tau was observed in the present study. Taurine is one of the most abundant metabolites in brain, and specifically has been shown to have a protective effect against excitotoxicity. Unlike GSH, the role of Tau is maintenance of calcium homeostasis, and in the context of excitotoxicity, protection against elevated levels of Ca^2+^ associated with GluR activation^45^. As such, Tau may parallel glutathione’s protective effects in relation to BMAA excitotoxicity (i.e., mitochondria-mediated oxidative stress). It is particularly notable that, like glutathione, Cys is the biosynthetic precursor for Tau; glial cells biosynthesize hypotaurine from Cys, and in turn, transport hypotaurine to neurons for subsequent synthesis of Tau^46^. The inhibition of the ${{\rm{X}}}_{{\rm{c}}}^{-}$ transporter by BMAA may, thus, not only decrease Cys availability for biosynthesis of GSH, but also Tau. Finally, in addition to its protective role, Tau is a co-activator of the inhibitory neurotransmitter, GABA, in the thalamus, and known to exert an anxiolytic effect in the brain. As such, Tau supresses neuroexcitatory activity including GluR stimulation such as that associated with excitotoxicity of BMAA. The decline in Tau represents, therefore, a novel – but, likely, important and multifaceted - contributor to BMAA neurotoxicity.

### Inhibition of Lipid Biosynthesis

An additional, novel branch of the metabolic network affected by BMAA, as revealed in the present study, is apparent attenuation of lipid biosynthetic pathways, and particularly those relevant to neurochemical networks, by BMAA exposure. Both FA and Chol were significantly decreased, in a dose-dependent manner, in embryos exposed to BMAA (Figs 2 and 4B). Neural cells - as the presumptive target of BMAA - are enriched in a diversity of lipids not found in any other tissue^47^. These lipids are essential to not only cell integrity (i.e., cell membrane components), but diverse neural functions. Among these roles, lipids – and particularly cholesterol - comprise up to 85% of protective myelin^48^. Phospholipids, on the other hand, have been shown to play a key role in both cell membrane integrity, and cell-signaling as it relates, in particular, to neuronal function (e.g., neurotransmitter release, regulatory roles)^47^.

Alongside decreased FA and Chol, a significantly dose-dependent decrease in NAA, as the key biosynthetic precursor for lipids, was measured. It is generally established that NAA (which is almost exclusively found in the neural cells) is primarily biosynthesized in the mitochondria of neurons, and transported, in turn, to oligodendrocytes where cleavage of NAA by aspartoacylase to acetate (and Asp), and subsequently acetyl CoA, provides biosynthetic building blocks for lipid synthesis including FA and Chol^49^. Mitochondrial dysfunction is, in fact, well linked with changes (i.e., reductions) in NAA levels in neural systems, and conversely, reductions in NAA are, in fact, utilized as a biomarker for mitochondrial disruption^50^. Moreover, there is growing evidence linking myelination to various neurogenerative diseases^51^. The observed effects of BMAA on NAA production, and subsequent lipid biosynthesis, therefore, might reveal a novel mechanism for contribution of BMAA to neurodegenerative disease.

Elaboration of lipid profiles by HPTLC/MALDI-ToF further reveal an intersection with alterations in two metabolites generally associated with biochemical modification of lipids. In the first case, although FA generally decreased, PE lipids were found (by HPTLC/MALDI-ToF) to increase, and the observed increase aligns with concurrent increase in EA^52,53^. Alongside phosphatidylcholine, PE are among the most abundant lipids in the brain where they function, as essential components of the plasma membrane, during neuronal differentiation including, in particular, neuritogenesis^54^. Enzymatic conversion of PE to PC via sequential methylation by phosphatidylethanolamine *N*-methyltransferase (PEMT), in turn, has been shown to be expressed in a regulated, stage-dependent manner during embryo development^55^. Recent studies demonstrate a role of PE in the mitochondrially mediated homeostastis of α-synuclein, as well as crosstalk between mitochondria and the endoplasmic reticulum, as it relates to neurodegeneration, and furthermore, show that EA supplementation can counter lipid disequilibrium in this regard^56,57^. It is, accordingly, proposed that increased PE (via upregulation of EA) may, therefore, represent a compensatory mechanism associated with BMAA effects on neural development. Notably, EA is directly biosynthesized from Ser, and upregulation of EA biosynthesis might, in turn, explain (alongside, or in addition to, a possible role of serinolysis, as discussed above) the reduced levels of Gly as a, likewise, direct biosynthetic product of Ser (Fig. 5).

Consistent, on the other hand, with apparently elevated glycolysis (i.e., metabolic reprogramming) in the treated embryo, a decrease in PI and m-Ins (an essential substrate for PI biosynthesis) was observed. The biosynthetic precursor of m-Ins is glucose-6-phosphate (G6P) which is, in turn, a key metabolic intermediate in the glycolytic pathway, and reduced inositol may, thereby, reflect redirection of G6P (toward Lac). Numerous, previous studies demonstrate a role of inositol-containing lipids (as substrates for kinases/phosphatatases) in various key regulatory functions in the brain^47^ including neural development. And moreover, emerging evidence suggests a contribution to both neuroexcitatory processes^58^, and neurodegeneration^59^. Taken together with the other diverse roles of lipids in cell membrane integrity, and myelination, observed effects on lipid biosynthesis (as revealed by the present integrated metabolomics approach) point to a previously unrecognized role of these metabolic pathways with respect to the apparently multifaceted contribution of BMAA to neurodegeneration.

## Methods

### Chemicals

All chemicals, including BMAA were obtained from (Sigma-Aldrich, St. Louis, MO, U.S.A.) unless otherwise mentioned.

### Zebrafish Embryo Breeding, Collection and Exposures

Husbandry and experimental procedures involving zebrafish embryos (i.e., exposures, and collection of embryos) were performed at the UFZ Helmholtz Centre for Environmental Research (Leipzig, Germany) in accordance with the German animal protection standards, and were approved by the Government of Saxony, Landesdirektion Leipzig, Germany (Aktenzeichen 75-9185.64). Adult wild-type zebrafish (*Danio rerio*) were maintained in recirculating aquarium systems according to established rearing procedures^19^. Breeding and embryo collection was performed by following the standard procedure as described earlier^60^ (see supplementary information). For BMAA exposures, embryos (either 3 or 72 hpf) were transferred to a sterile 6-well tissue culture plate, and exposed to two concentrations of BMAA (85 µM and 170 µM, i.e., BMAA_85_ and BMAA_170_), for 24 hours. Subsequently the embryos were collected and carefully washed three times with MilliQ water to remove any residual BMAA. The embryos were either directly used for HR-MAS NMR analysis or stored at −80 °C until analysis.

### Visualization of ROS

Intracellular ROS generation in whole zebrafish embryos was visualized using the probe chloromethyl-2′,7′-dihydrodichlorofluorescein diacetate (CM-H_2_DCFA; Molecular Probes) as a nonfluorescent cell-permeative compound. Intracellular esterases cleave the acetate groups such that the nonfluorescent dye 2′,7′-dichlorofluorescein (DCF) is retained intracellularly, which, in turn, is oxidized by intracellular ROS and thus becomes fluorescent. At 96 hpf, CM-H_2_DCFA (1 mM solution in 4% DMSO) was added to control and BMAA_170_ treated embryos in egg water to a final concentration of 10 μM, and incubated for another 60 min. Subsequently, embryos were washed 3 times with egg water to remove excess CM-H_2_DCFA in the medium. Whole embryos were observed under microscope (DM IRB; Leica, Wetzlar, Germany), equipped with a 10x objective lens and differential interference contrast (DIC) optics. DIC images were acquired to document the bright field (BF) image of the whole embryos. The fluorescent product DCF was detected with an excitation wavelength of 485 nm, and emission wavelength of 530 nm, and images were obtained in FITC mode. Post-acquisition analysis was performed with Corel Photo-Paint X6 software with minimal alterations to background and contrast, which were applied equally to samples and controls.

### Quantification of Glutathione (GSH)

The embryo extracts were prepared in 5% 5-sulfosalicylic acid and subjected to GSH quantification using GSH assay kit according to manufacturers’ instruction (Sigma-Aldrich). Control and BMAA_170_ treated extracts (250 μL) from 96 hpf embryos were mixed with 250 μL of working solution which consist of reaction buffer, diluted GSH reductase (enzyme) and 5,5′-dithiobis-2-nitrobenzoic acid (DTNB). This reaction produces GSH disulfide (GSSH) and 5-thio-2-nitrobenzoic acid (TNB) which is a yellow product measured photometrically at 412 nm. Fifty microliters of this solution were added to the 96-well plates, and incubated at 37 °C for 10 minutes. After this step, 50 μL of diluted NADPH stock solution in reaction buffer were added to each well plate, and incubated again for 20 minutes at 37 °C. In the presence of NADPH, GSH reductase converts GSSG to GSH which is utilized in the previous reaction and produces even more TNB. Therefore, this recycling reaction improves the sensitivity of total GSH detection. The absorbance of each well was measured using a multiplate fluorimeter (Tecan Infinite 200 Pro, Männerdorf, Switzerland).

### Measurement of Cholesterol

The extraction of lipid and metabolite layer from zebrafish embryos was performed as described previously^19^. The embryos (100 embryos) were crushed in 1 mL methanol:water (1:1, v/v) mixture. Subsequently, 1 mL chloroform was added. The mixture was then sonicated for 15 min and centrifuged at 5000 rpm at 4 °C. After centrifugation, the two layers (lower chloroform layer and upper methanol:water layer) were carefully separated, and each was dried individually under nitrogen gas flow at 4 °C. The dried non-polar lipids were subjected to cholesterol quantification using an Amplex Red Cholesterol Assay Kit (Invitrogen, Carlsbad, CA) according to the manufacturer’s instructions. Control and BMAA_170_ treated apolar lipids from 96 hpf embryo were suspended in 500 µL of methanol. Five microliters of these solutions were diluted in 45 µL of the reaction buffer (1∶10) and added to a 96-well black microplate. Subsequently, 50 µL of a solution containing Amplex Red Reagent/Horseradish Peroxidase (HRP)/cholesterol oxidase/cholesterol esterase were added to every well and the reaction was incubated for 30 minutes, at 37 °C, protected from light. The reaction buffer (without cholesterol) alone or containing 10 mM of H_2_O_2_ were used as negative and positive controls, respectively. The fluorescence levels were measured using a multiplate Fluorimeter (Tecan Infinite 200 Pro, Männerdorf, Switzerland) using 560 nm for excitation and 590 nm for emission.

### MALDI-TOF of Lipids

The lipid extract from zebrafish embryos was subjected to high-performance thin-layer chromatography (HPTLC) prior to MALDI-TOF MS. MALDI-TOF mass spectra were acquired using an Autoflex I mass spectrometer (Bruker Daltonics) with ion reflector, as described previouslty^17,18^. The details of experimental procedure of MALDI-ToF mass spectrometry coupled to thin-layer chromatography (TLC) are provided in supplementary information.

### HR-MAS NMR

For HRMAS analysis, 100 embryos were carefully inserted into a 4-mm zirconium oxide rotor for each of the 9 measurements (n = 9) per group. As NMR reference (1H chemical shift at 0 ppm), 10 µL of deuterated phosphate buffer (100 mM; pH 7.0), containing 0.1% (w/v) 3-trimetylsilyl-2,2,3,3-tetradeuteropropionic acid (TSP) was added to each, and the rotor was placed immediately inside the NMR spectrometer.

All HR-MAS NMR experiments were carried out on a Bruker DMX 600 MHz NMR spectrometer operating with a proton resonance frequency of 600 MHz. The instrument is equipped with a 4-mm HRMAS dual inverse ^1^H/^13^C probe with a magic-angle gradient. All measurements were carried out at a magic-angle spinning rate of 6 kHz, and a temperature of 277 K. Temperature was controlled by a Bruker BVT3000 control unit. Bruker TOPSPIN software (Bruker Analytische Messtechnik, Germany) were used to acquire and process the NMR data.

One-dimensional ^1^H HR-MAS NMR spectra were recorded using rotor synchronized Carr-Purcell-Meiboom-Gill (CPMG) pulse sequence with water suppression. Each one-dimensional spectrum was acquired applying a spectral width of 8000 Hz, domain data points of 16k, number of averages of 512 with 8 dummy scans, constant receiver gain of 2048, and acquisition time of 2 s and relaxation delay of 2 s. Since NMR measurements were done on intact embryos, the relaxation delay was set to a small value to remove short *T*_2_ components due to the presence of lipids. All spectra were processed by an exponential window function corresponding to a line broadening of 1 Hz and zero-filled before Fourier Transformation. NMR spectra were phased manually, and automatically baseline corrected using TOPSPIN 2.1 (Bruker Analytische Messtechnik, Germany). The total analysis time (including sample preparation, optimization of NMR parameters and data acquisition) of ^1^H-HRMAS NMR spectroscopy for each sample was approximately 20 min.

To confirm the signal assignments, 2D homo-nuclear correlation spectroscopy (¹H-¹H COSY) in magnitude mode was performed using Bruker’s standard pulse program library. The parameters used for COSY were 2048 data points collected in the *t*_2_ domain over the spectral width of 4k, 512 *t*_1_ increments were collected with 16 transients, relaxation delay 2 sec, acquisition time 116 msec, and pre-saturated water resonance during relaxation delay. The resulting data were zero filled with 2048 data points, and were weighted with sine bell window functions in both dimensions prior to Fourier Transformation. To confirm that there is no sample degradation during ^1^H-^1^H COSY experiment, we measured 1D ^1^H HRMAS spectra before and after ^1^H-^1^H COSY measurements (Supplementary Fig. S2).

Two-dimensional *J-*resolved measurements in magnitude mode was performed with control 96 hpf embryo using Bruker’s standard HRMAS pulse program library (jresgpprgf for “*J-*resolved” experiment). NMR signals from *J* coupled protons are always J modulated. *J* values of metabolites are in the range of 0–20 Hz. To visualize the *J* coupling based splitting of the NMR signal, 21k data points were collected in the *t*_2_ time domain and 64 data points in *t*_1_ time domain. Spectral width for *t*_2_ domain was 9 kHz and 50 Hz for *t*_1_ domain. No of increments were 128 and dummy scans were 8. The spectra were zero filled with 2048 data points in each dimension. This allows the 2D spectra to split row wise in 2048 individual spectra and each of such spectra contains the information of *J* coupling and the metabolites contributing.

In addition, further confirmation of assignment was achieved by comparing HRMAS NMR spectra of intact embryos with liquid state NMR spectra obtained from the metabolites extracted from embryos (see supplementary information for method details) (Supplementary Fig. S15). The chemical shift assignments were further compared with the online HMDB database (http://www.hmdb.ca).

### ^1^H NMR Data Analysis

All of the spectra were referenced, baseline-, phase-corrected and analysed by using MestReNova v.8.0 (Mestrelab research S.L., Spain). Quantification of metabolites was performed using Chenomx NMR Suite 8.3 which allowed for qualitative and quantitative analysis of an NMR spectrum by fitting spectral signatures from an HMDB database to the spectrum. Metabolite concentrations were subsequently calculated as ratio to tCr. Since external reference might lead to the misleading results, Cr resonance is a reliable internal reference widely used in animal studies. One-way analysis of variance (ANOVA) of the NMR quantification results were performed with OriginPro v. 8 (Northampton, USA). F-values were calculated, and F-values larger than 2.8 (p < 0.05) were considered significant.

Quantification of Glc and EA was done from 2D spectra as described in supplementary information.

### Data availability

All data generated or analysed during this study are included in this published article (and its Supplementary Information files). Additional raw data files can be available from the corresponding author on request.

## Electronic supplementary material

Supporting Information
