# Supplementary material for: Metabolic profiling of zebrafish (Danio rerio) embryos by NMR spectroscopy reveals multifaceted toxicity of β-methylamino-L-alanine (BMAA)
Source: Sci Rep. 2017 Dec 11;7:17305. doi: 10.1038/s41598-017-17409-8 (PMC5725574; doi:10.1038/s41598-017-17409-8)
Supplement: Supplementary file 1 — Supporting Information [file 41598_2017_17409_MOESM1_ESM.doc]

Supporting Information

**Metabolic profiling of zebrafish (*Danio rerio)* embryos by NMR spectroscopy reveals multifaceted toxicity of β-methylamino-L-alanine (BMAA)**

Upasana Roy^1,3^, Laura Conklin^2^, Jürgen Schiller^1^, Jörg Matysik^3^, John P. Berry^2*^ and A. Alia^1,4*^

^1^Institute for Medical Physics and Biophysics, University of Leipzig, D-04107 Leipzig, Germany;

^2^Department of Chemistry and Biochemistry, Florida International University, North Miami, FL 3318, USA;

^3^Institute of Analytical Chemistry, University of Leipzig, D-04103 Leipzig, Germany;

^4^ Leiden Institute of Chemistry, 2333 Leiden, The Netherlands

**Methods**

**Zebrafish Embryo Breeding, Collection and Exposures.** Adult wild-type zebrafish (*Danio rerio*) were maintained in recirculating aquarium systems according to established rearing procedures^1^. The water temperature was maintained at 28ºC with a flow rate of 150 L/min, with day/night light cycles (12h dark versus 12h light). The fish were fed twice daily with commercial flake food according to the guidelines for the laboratory use of zebrafish^2^. All fish were handled according to Institutional Animal Care and Use Committee (IACUC) guidelines.

Breeding and embryo collection was performed by following the standard procedure as described earlier^3^. Briefly, eggs were obtained by random pairwise mating of zebrafish. Three adult males and four females were placed together in small breeding tanks (Ehret GmbH, Emmendingen, Germany) the evening before eggs were required. The breeding tanks (L 26 cm; H 12.5 cm; W 20 cm) contain mesh egg traps to prevent the eggs from being eaten. The eggs were harvested the following morning, and transferred into 92-mm plastic Petri dishes (50 eggs per dish) containing 40 mL fresh embryo buffer as described^4^. Eggs were washed four times to remove debris, while unfertilized, unhealthy and dead embryos were removed under a dissecting microscope. At 3.0 hpf, embryos were again screened and any further dead and unhealthy embryos were removed. Throughout all procedures, the embryos and the solutions were kept at 28°C, either in the incubator or a climatized room. All incubations of embryos were carried out in an incubator with orbital shaking (50 rpm) under a light cycle of 14 h light: 10 h dark (lights on at 8 h).

**MALDI-TOF of Lipids.** For the extraction of lipids from zebrafish embryos a modified procedure of Suhartono et al.^4^ was used. The embryos (100 embryos) were crushed in 1 ml methanol:water (1:1, v/v) mixture. Subsequently, 1 mL chloroform was added. The mixture was then sonicated for 15 min and centrifuged at 5000 rpm at 4°C. After centrifugation, the two layers (lower chloroform layer and upper methanol:water layer) were carefully separated and each was dried individually under nitrogen gas flow at 4°C. The dried chloroform layer containing the lipids was dissolved in 1 ml deuterated chloroform, and subsequently filtered using a Millipore filter (Millex-HV 0.45 μm Filter Unit).

Prior to MALDI-TOF MS, the organic extract from zebrafish embryos was subjected to high-performance thin-layer chromatography (HPTLC) as described previously^1,5,6^. Sample were applied on to HPTLC silica gel 60 plates (10x10 cm^2^ with aluminum backs; Merck, Darmstadt, Germany), and developed in vertical TLC chambers using CHCl_3_–ethanol–water–triethylamine (35/35/7/35, v/v/v/v) as the mobile phase for the separation of phospholipids. Lipids were visualized by spraying with a solution of primuline^7^. Upon excitation by ultraviolet light (366 nm), individual lipids become detectable as colored spots. These spots were assessed by using a digital image system in combination with the program Argus X1 delivered by BioStep (Jahnsdorf, Germany).

For MS analysis, TLC plates were cut into smaller pieces containing all relevant lipid spots. These pieces were mounted to a prototype MALDI-TOF-MS adapter target^8^ with double-sided, conductive adhesive tape (Leit Tabs, Plano GmbH, Wetzlar, Germany). All MALDI spectra of TLC-separated lipids were directly obtained from the HPTLC plates as described in more detail previously ^8^. The matrix addition (a 100 mg/mL solution of 2,5-dihydroxybenzoic acid [DHB] in acetonitrile–water, 1:1 v/v) was performed manually on circled spots monitored by previous primuline staining. Selected negative ion mass spectra were recorded in the presence of 9-aminoacridine as recently described ^9^.

All MALDI-TOF mass spectra were acquired using an Autoflex I mass spectrometer (Bruker Daltonics) with ion reflector, as described previouslty ^5,6^ . The system utilizes a pulsed 50 Hz nitrogen laser emitting at 337 nm. The extraction voltage was 20 kV, and gated matrix suppression was applied to prevent the saturation of the detector by matrix ions. All spectra were acquired in the reflector mode using delayed extraction. Spectral mass resolutions and signal-to-noise ratios were determined by the instrument software Flex Analysis 2.4 (Bruker Daltonics). The mass spectrometer was calibrated using the molecular ions of a lipid mixture desorbed from a standard DHB preparation applied next to the spots of interest.

**^1^H Liquid NMR.** The metabolites from 96 hpf embryos were extracted according to the method described earlier^1^. The NMR spectra of the metabolite layer were recorded with a Bruker 400 MHz NMR spectrometer using a 5mm inverse triple high-resolution probe with actively shielded gradient coil. The ^1^H NMR spectra were accumulated with 65,000 data points, a 1.5 s relaxation delay, and a sweep width of 12.4 kHz; 256 scans were required to obtain a satisfactory signal to noise ratio. The free induction decays (FIDs) were weighted with an exponential function (0.09 Hz) before Fourier transformation.

**Quantification of glucose and ethanolamine from 2D spectra.** Quantification of glucose and ethanolamine were done by integrating cross peaks of glucose (3.23, 4.63ppm) and ethanol amine (3.14, 3.83ppm) in 2D spectra and multiplying by a factor which was obtained by comparing the ratio of the peak area of other well resolved metabolite (*Z*) to peak area of creatine (tCr). *Z is* a metabolite which has well resolved signals in both 1D and 2D (e.g. Tau). Following equations were used:


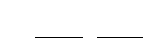


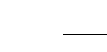


*m*=multiplication factor; *A*=area under the peak (1D) or cross peak (2D); Z= metabolite which have well resolved signal in both 1D and 2D; *C_x_*= concentration relative to creatine of the metabolite of interest.


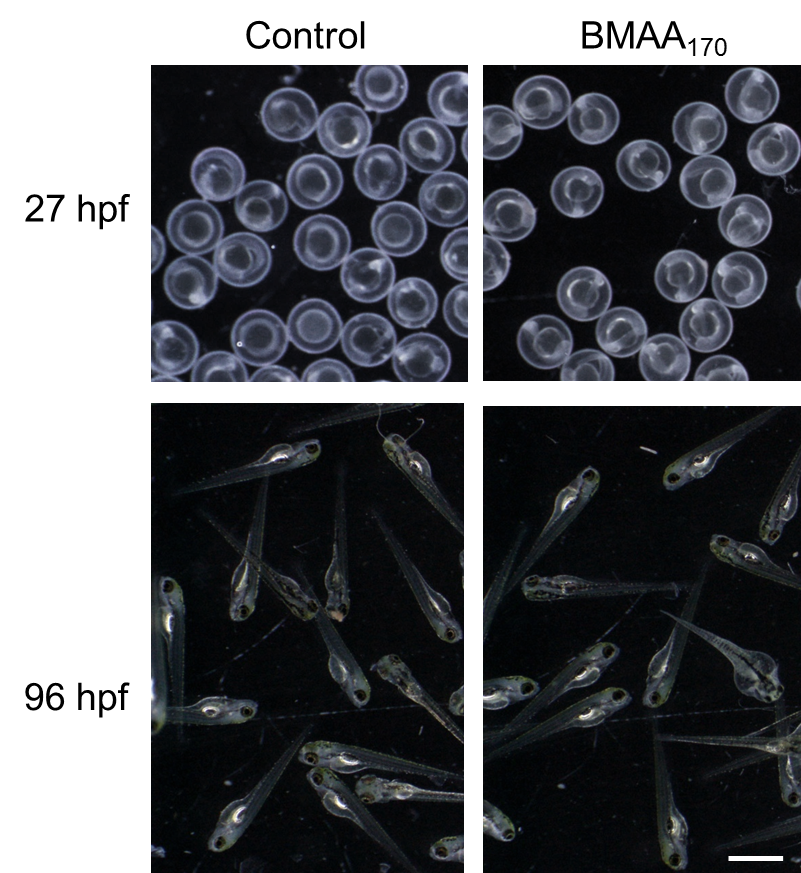


**Supplementary Fig. S1.** Light microscope images of 27 hpf and 96 hpf Zebrafish embryos after treatment without (control) or with BMAA_170_ for 24 hours. Scale bar: 1.0 mm.


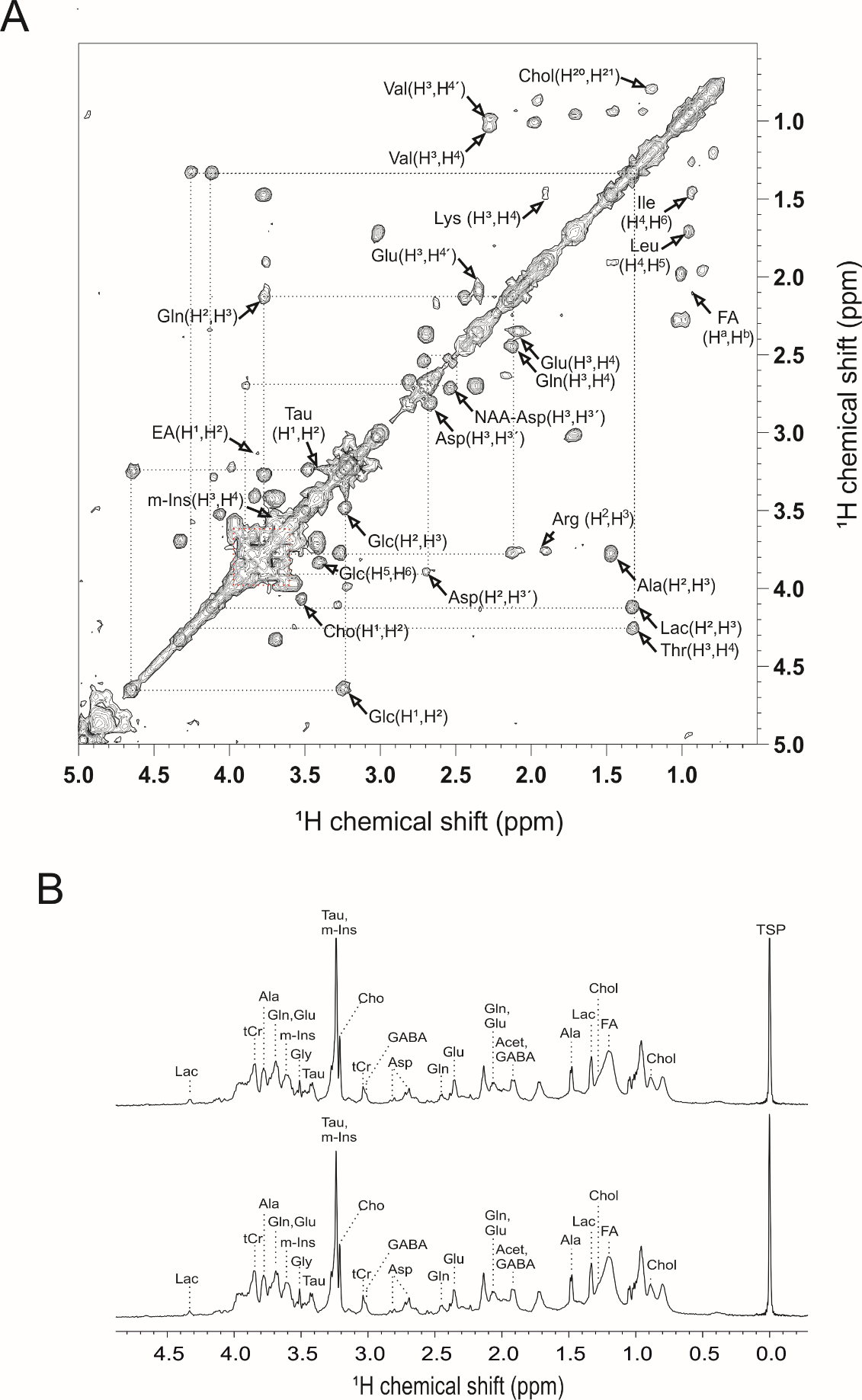


**Supplementary Fig. S2. (A)** ^1^H-^1^H COSY spectra for control 27 hpf embryo were recorded in magnitude mode. The parameters used for COSY were 2048 data points collected in the t2 domain over the spectral width of 4k, 512 t1 increments were collected with 16 transients, relaxation delay 2 sec, acquisition time 116 msec, and pre-saturated water resonance during relaxation delay. The resulting data were zero filled with 512 data points, and were weighted with the squared sine bell window functions in both dimensions prior to Fourier Transformation. Application of gradient pulses along with tradition ^1^H-^1^H COSY sequence provides resolution compared to liquid NMR. Several other metabolites were observed which were not unambiguously detected in the 1D spectra; e.g. Val, Ile, Leu, Arg, Lys. (B) ^1^H HRMAS spectra of control 27 hpf zebrafish embryo recorded before (upper) and after (lower) ^1^H-^1^H COSY measurements. No sign of metabolite degradation was observed in the spectra.


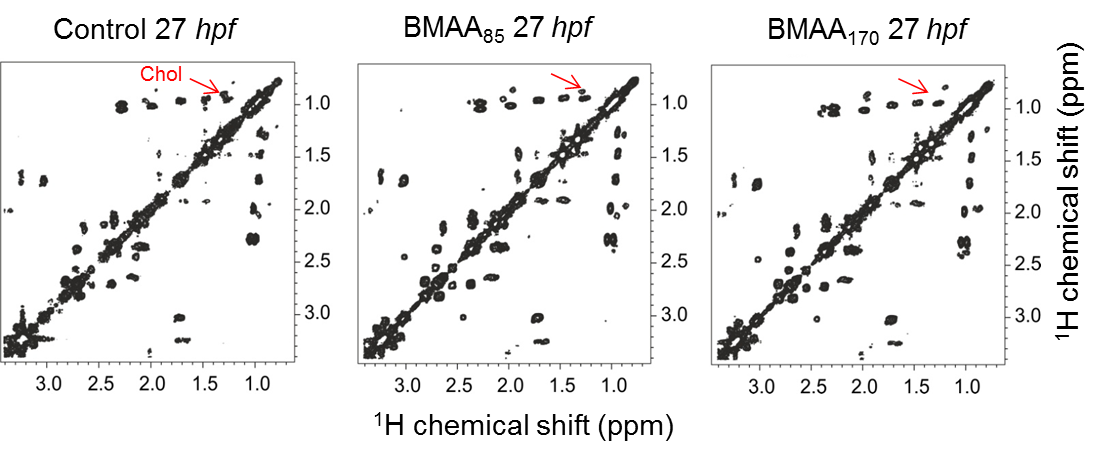


**Supplementary Fig. S3.** . Comparison of ^1^H-^1^H COSY spectra of 27 hpf embryos of control, BMAA_85_ and BMAA_170_ treated embryo in the range of 1.0 to 3.0 ppm. Crosspeak from Chol is reduced with BMAA treatment.


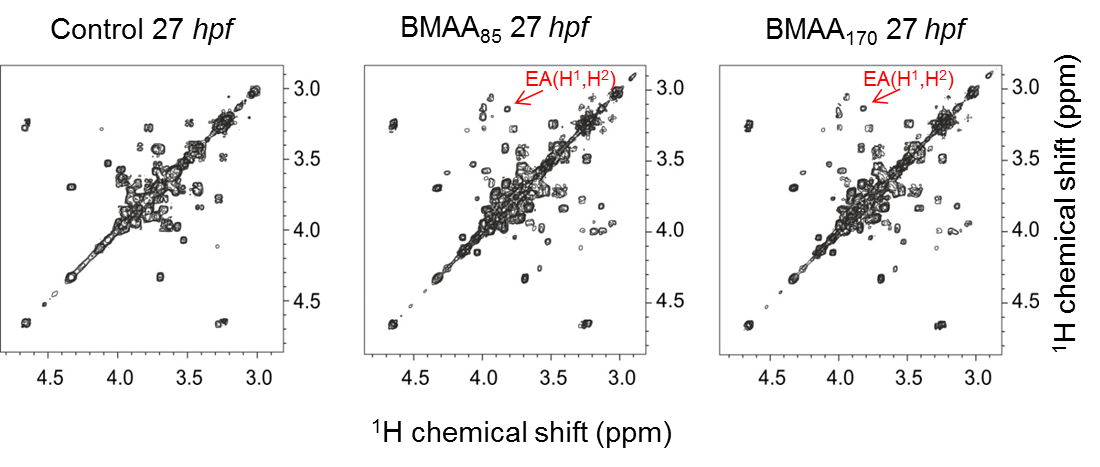


**Supplementary Fig. S4.** Comparison of ^1^H-^1^H COSY spectra of 27 hpf embryos of control, BMAA_85_ and BMAA_170_ treated embryo in the range of 3.0 to 4.5 ppm. Crosspeak from EA is increased with BMAA treatment.


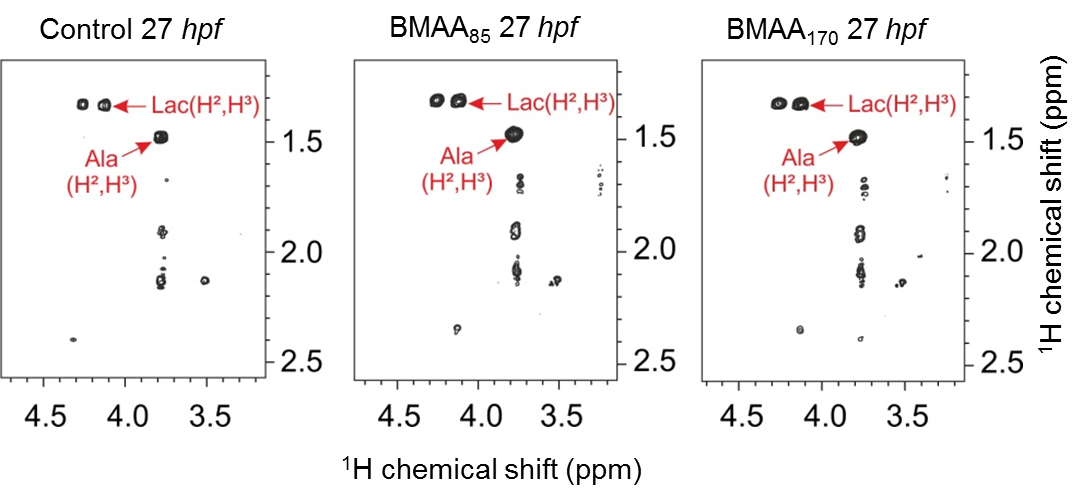


**Supplementary Fig. S5.** Comparison of ^1^H-^1^H COSY spectra of 27 hpf embryos of control, BMAA_85_ and BMAA_170_ treated embryo in the range of 1.5 to 2.5 and 3.5 to 4.5 ppm. Cross peaks from Lac and Ala are increased with BMAA treatment in a dose dependent manner.


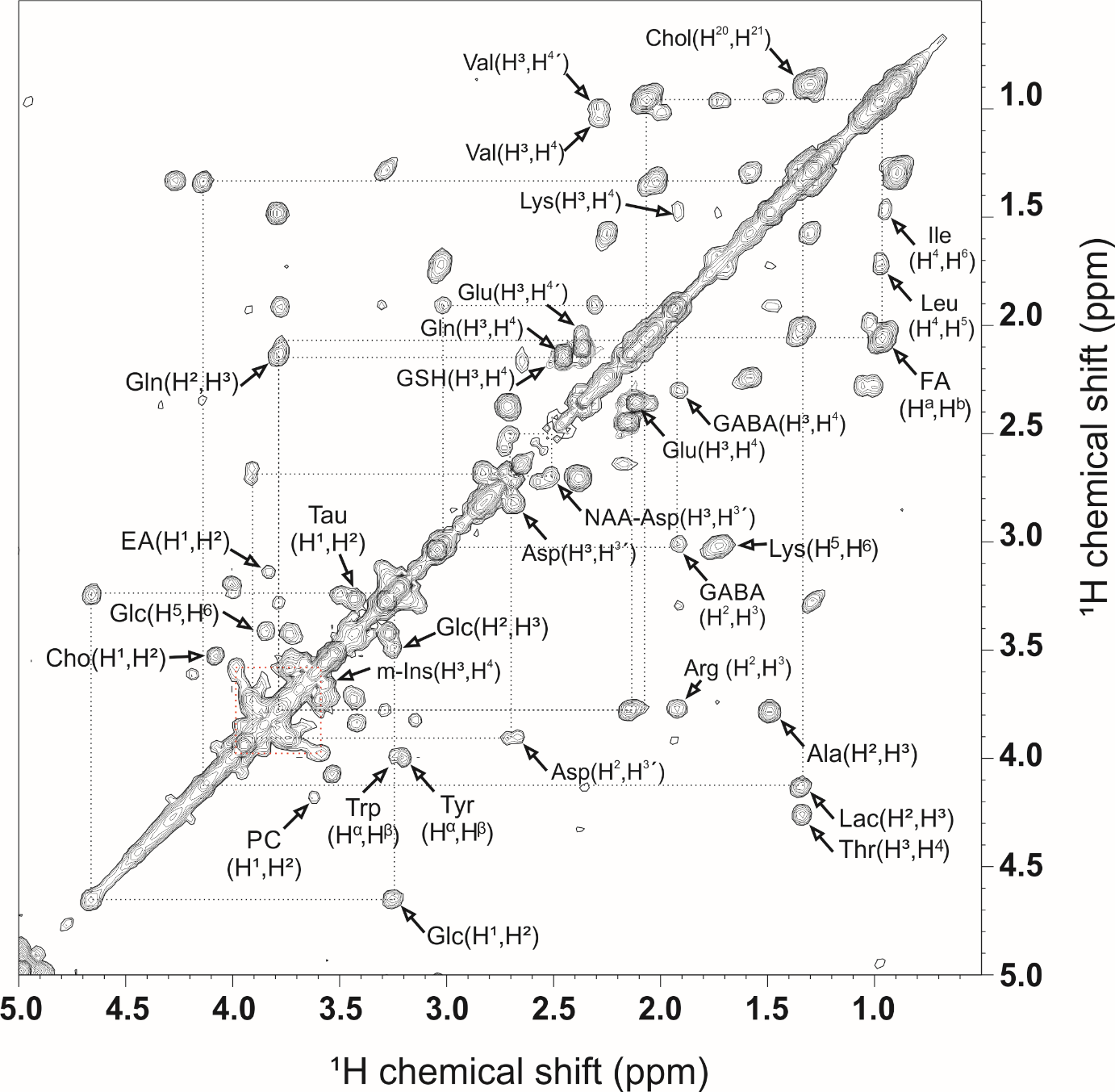


**Supplementary Fig. S6.** ^1^H-^1^H COSY spectra for control 96 hpf embryo were recorded in magnitude mode. The parameters used for COSY were 2048 data points collected in the t_2_ domain over the spectral width of 4k, 512 t_1_ increments were collected with 16 transients, relaxation delay 2 sec, acquisition time 116 msec, and pre-saturated water resonance during relaxation delay. The resulting data were zero filled with 512 data points, and were weighted with the squared sine bell window functions in both dimensions prior to Fourier Transformation. Application of gradient pulses along with traditional ^1^H-^1^H COSY sequence provides resolution compared to liquid NMR. Like 27 hpf control several other metabolites were observed which were not unambiguously detected in the 1D spectra, e.g. Val, Ile, Leu, Tyr, Trp, Arg and Lys.


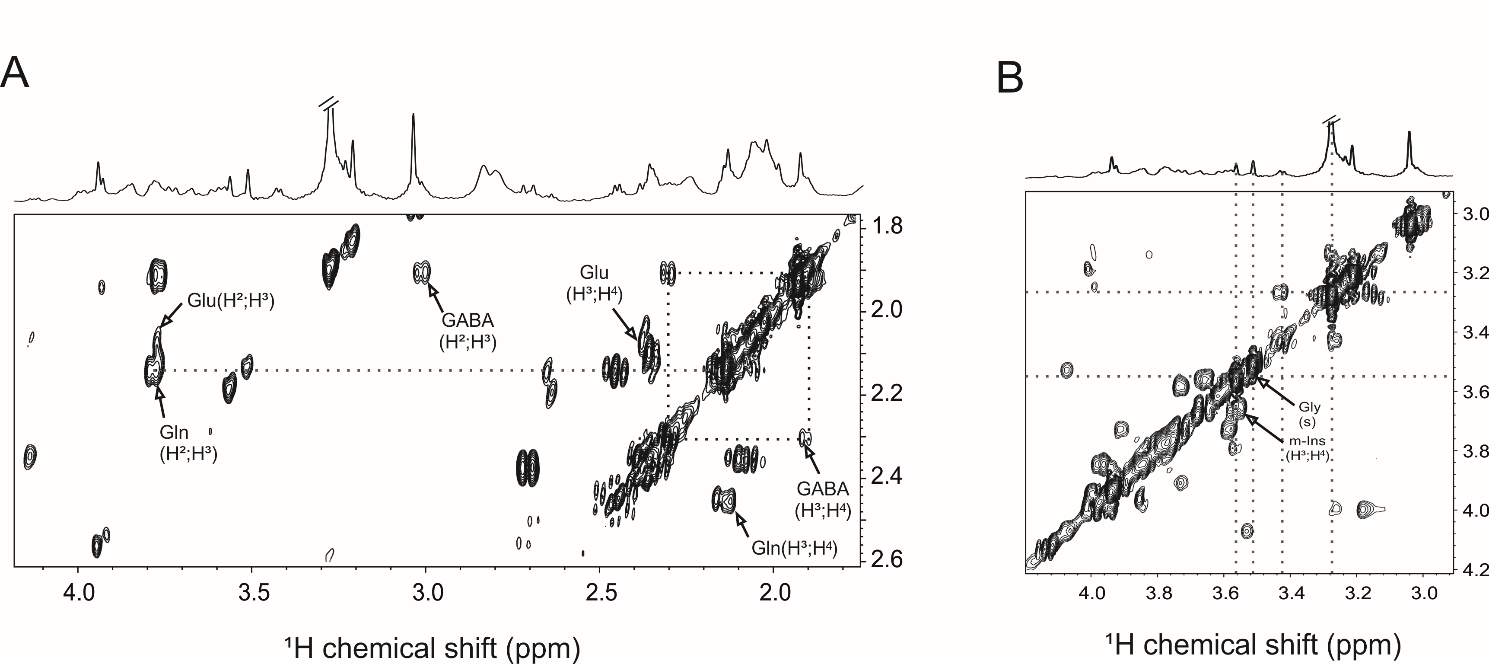


**Supplementary Fig. S7. (A)** ^1^H-^1^H COSY spectra for control 96 hpf embryo were recorded in magnitude mode showing (A) clear separation of crosspeaks of Glu, Gln and GABA confirming the 1D spectral assignment. (B) Zoomed region of ^1^H-^1^H COSY spectra highlighting the crosspeaks of Gly and m-Ins confirming the 1D spectral assignments


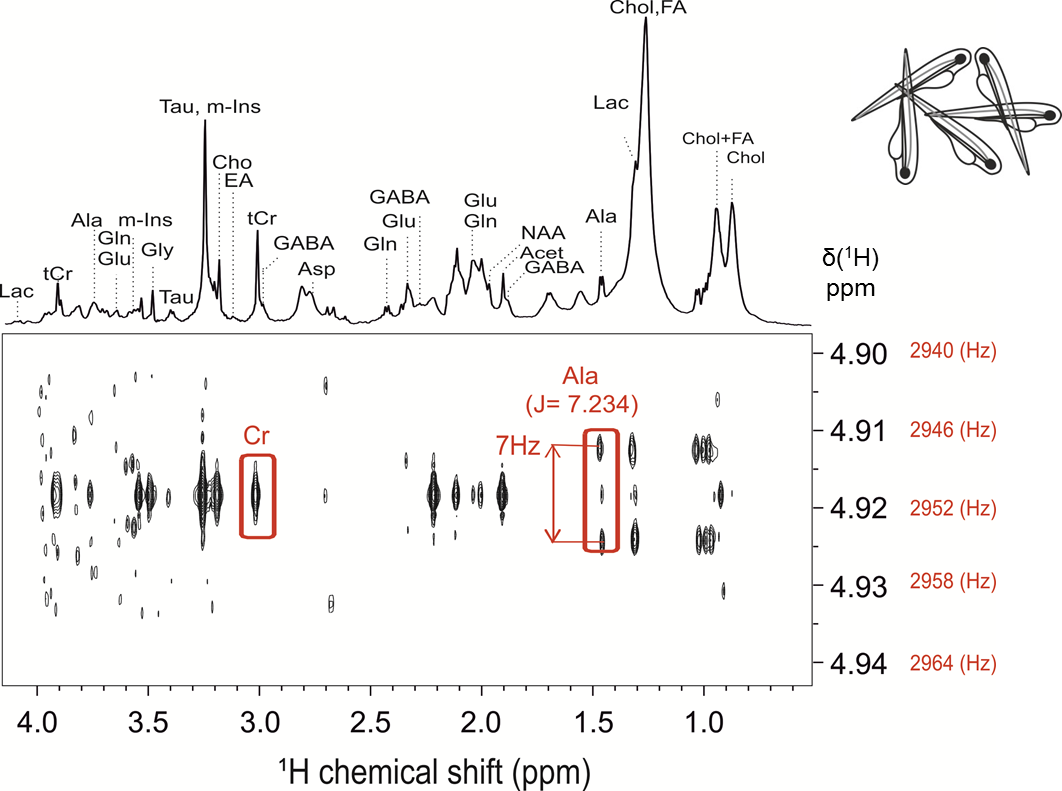


**Supplementary Fig. S8.** 2D *J-*resolved measurements in magnitude mode was performed with control 96 hpf embryo using Bruker’s standard HRMAS pulse program library (jresgpprgf for "*J-*resolved" experiment). NMR signals from J coupled protons are always *J* modulated. *J* values of metabolites are in the range of 0-20 Hz. To visualize the J coupling based splitting of the NMR signal, 20k data points were collected in the t_2_ time domain and 64 data points in t_1_ time domain. Spectral width for *t_2_* domain was 9 kHz and 50 Hz for *t_1_* domain. No of increments were 128 and dummy scans were 8. The spectra were zero filled with 2048 data points in each dimension. This allows the 2D spectra to split row wise in 2048 individual spectra and each of such spectra contains the information of *J* coupling and the metabolites contributing.


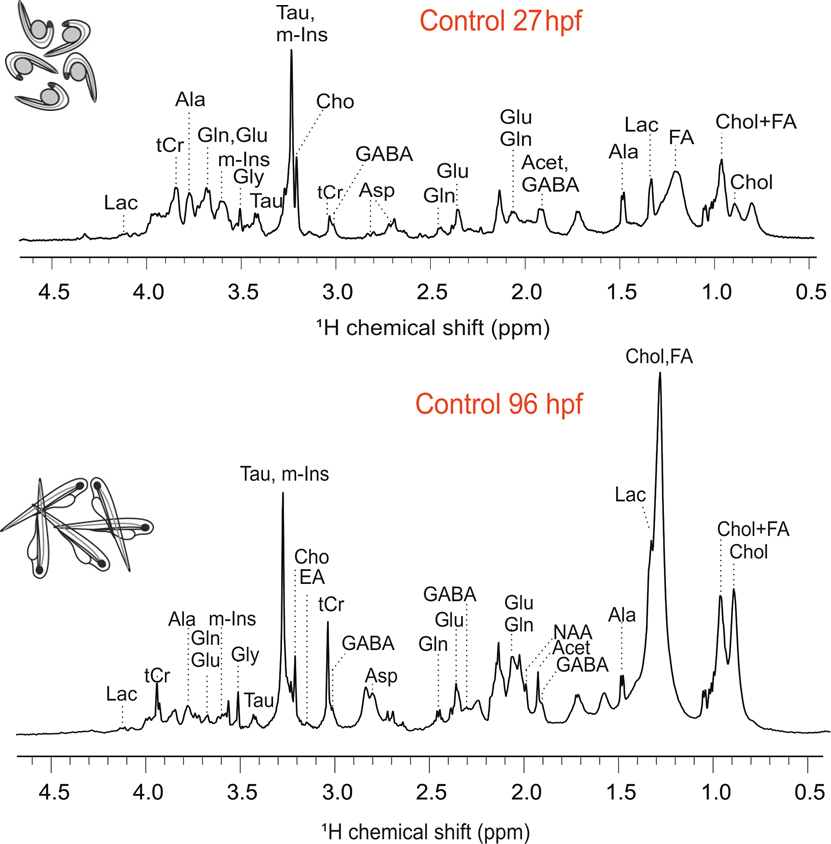


**Supplementary Fig. S9.** ¹H HRMAS NMR spectra showing metabolic profile of control 27 hpf and 96 hpf Zebrafish embryos. Interestingly, fatty acid synthesis was largely increased from 27 hpf to 96 hpf embryo. More creatine were produced in 96 hpf embryo linked with physiological development. At 27 hpf, blood islands, otoliths and midbrain-hindbrain boundary are already developed in the embryo and by 96 hpf details of CNS development is achieved including formation of telencephalon, mesencephalon, hypothalamus and, importantly, formation of primary and secondary motor neurons suggesting that spinal cord and brain is in a fast-developing stage at 96 hpf.


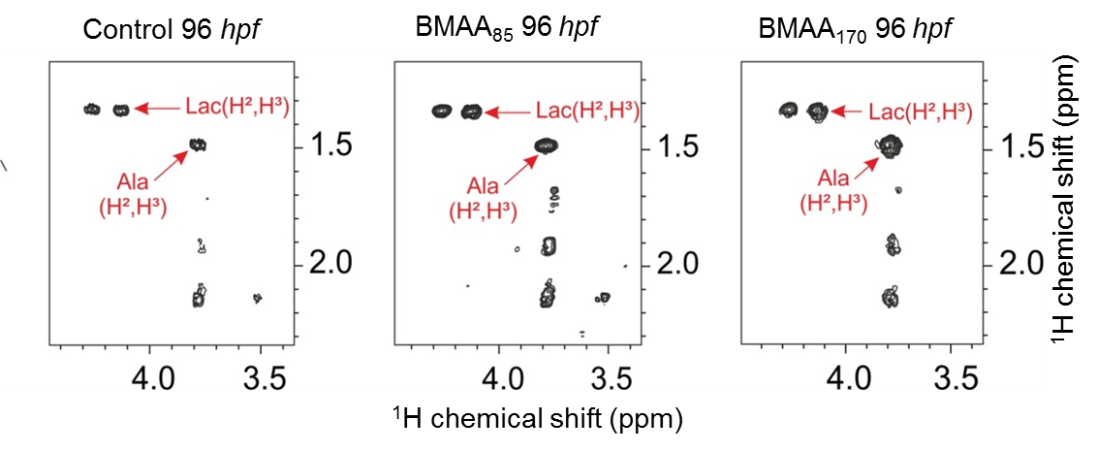


**Supplementary Fig. S10.** Comparison of ^1^H-^1^H COSY spectra of 96 hpf embryos of control, BMAA_85_ and BMAA_170_ treated embryos in the range of 1.5 to 2.5 and 3.5 to 4.5 ppm. Cross peaks from Lac and Ala are increased with BMAA treatment in a dose dependent manner.

**Multivariate statistical analysis**

Multivariate statistical analysis of primary metabolites in the spectra was performed using Bruker software package AMIX (version 3.8.6). The CPMG spectra, collected from embryos, were subdivided in the range between 0.3 and 9 ppm into buckets of 0.04 ppm (total 218 buckets), using Bruker AMIX software (Version 3.8.7, Bruker GmbH). The region of 4.80 – 6.00 ppm was excluded from the analysis to remove the water signal. To compensate for the differences in the overall metabolite concentration between individual samples, the data obtained were mean centered, scaled to unit variance and then normalized by dividing each integral of the segment by the total area of the spectrum. The resulting data matrix was exported into Microsoft office Excel (Microsoft Corporation, USA). This was then further imported into SIMCA software (Umetrics AB) for multivariate statistical analysis. A probability of p-value of <0.05 was taken to indicate statistical significance.


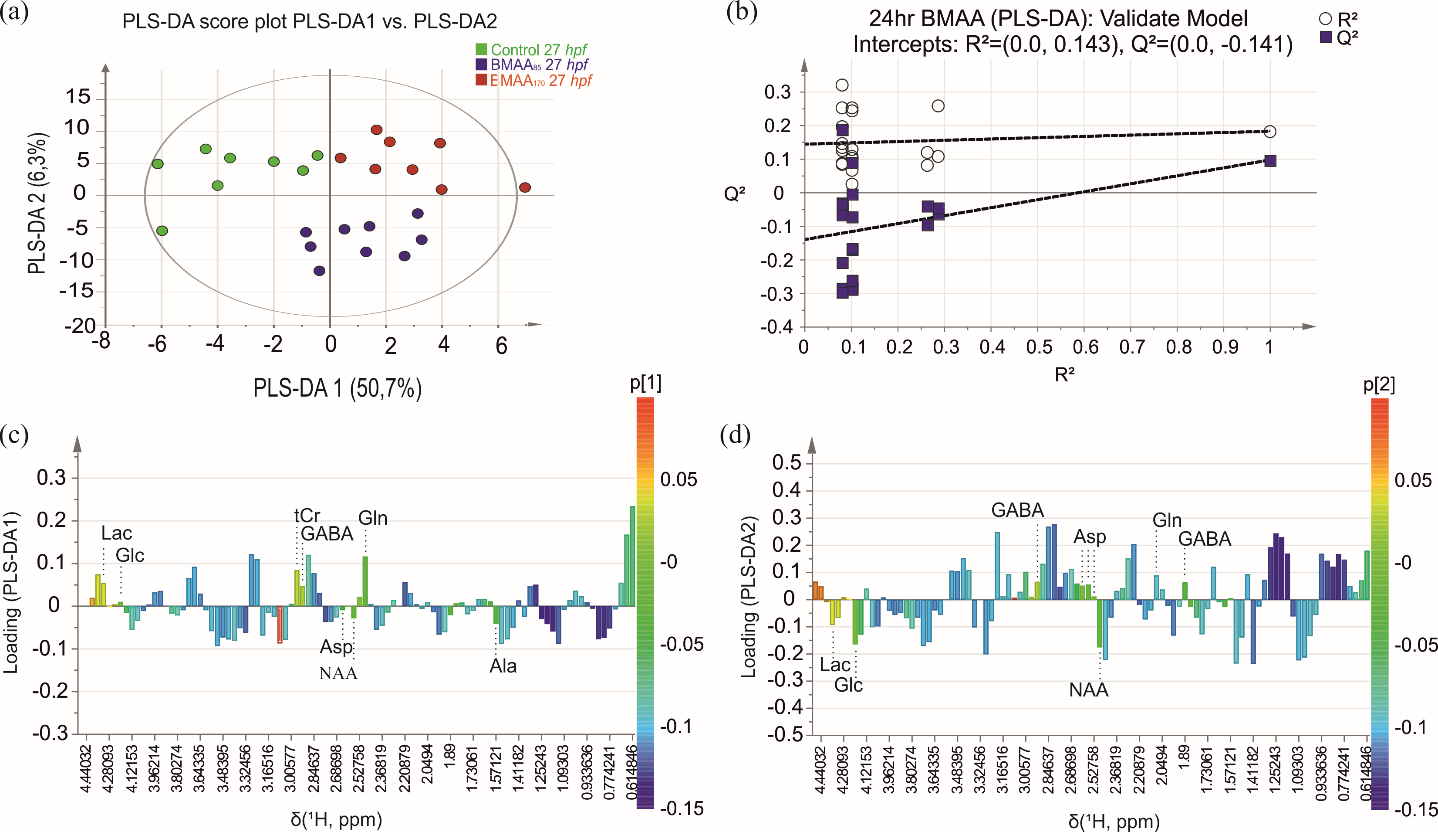


**Supplementary Fig. S11.** (a) PLS-DA scores plots for control 27 hpf, BMAA_85_ treated and BMAA_170_ treated embryo. Spectra derived from the same brain tissue have the same color. Total 57% variables are used to make this score plot. (b) Model validation plot with R^2^ positive intercepts at (0.0, 0.143) and Q^2^ negative intercepts at (0.0, -0.141). (c) Loading plots of PLS-DA1 and (d) Loading plot of PLS-DA2 for all buckets containing assigned peaks. GABA and Gln showed positive loading and NAA showed negative loading for both PLS-DA1 and PLS-DA2 indicating the most consisted metabolite for cluster separation in the score plot.

**PLS-DA analysis 24 hr embryo:** The two most popular methods for multivariate analysis are unsupervised principal component analysis (PCA) and supervised partial least squares discriminant analysis (PLS-DA). These methods give both a score matrix and a loading matrix, with score matrix showing the relation between observations, while the loading matrix gives the individual contribution of each parameter, which is a peak in the case of NMR spectra. The HRMAS spectra were investigated by multivariate analysis to probe if control and BMAA treated embryos can be discriminated and to determine the spectral regions and corresponding compounds mainly responsible for the separation. The PLS-DA scores plot of the of first two principle components explaining 57% of the total variance is shown in SIFig. 11(a). Even though not completely separated, a clear clustering could be observed in the score plot of PLS-DA1 vs PLS-DA2. The control and BMAA_170_ were separated by PLS-DA1 scores; whereas the control and BMAA_170_ treated embryos were separated from BMAA_85_ treated embryos by their PLS-DA2 scores. Control embryos were clustered towards the negative PLS-DA1 scale and BMAA_170_ treated embryos were clustered towards positive PLS-DA1 scale. Model validation plot with R^2^ positive intercepts at (0.0, 0.143) and Q^2^ negative intercepts at (0.0, -0.141) is shown in SI Fig. 11(b). In order to determine the variables, i.e. metabolites assigned to the corresponding buckets that are mainly responsible for the separation of three groups, the load values of the PLS-DA1 and PLS-DA2 are given in SI Fig. 11(c) and (d). Signals coming from Cr, Gln, GABA, Lac, Glc has positive score in the PLS-DA1 loading plot within 95% significance interval (*P*<0.05) and signal coming from Asp, NAA and Ala has negative score in the in the PLS-DA1 loading plot within 95% significance interval (*P*<0.05). Thus PLS-DA1 loading separates the control from BMAA_85_ and BMAA_170_ treated samples and the metabolites responsible for it is also showed the changes upon detection and quantification in the NMR experiments. Loading from PLS-DA2 also shows positive scores for GABA, Asp and Gln and negative score for Lac, Glc, NAA and Glu within 95% confidence level (*P*<0.05), thus making it responsible for separation of clusters for control and BMAA_85_ treated samples with BMAA_170_ treated samples.


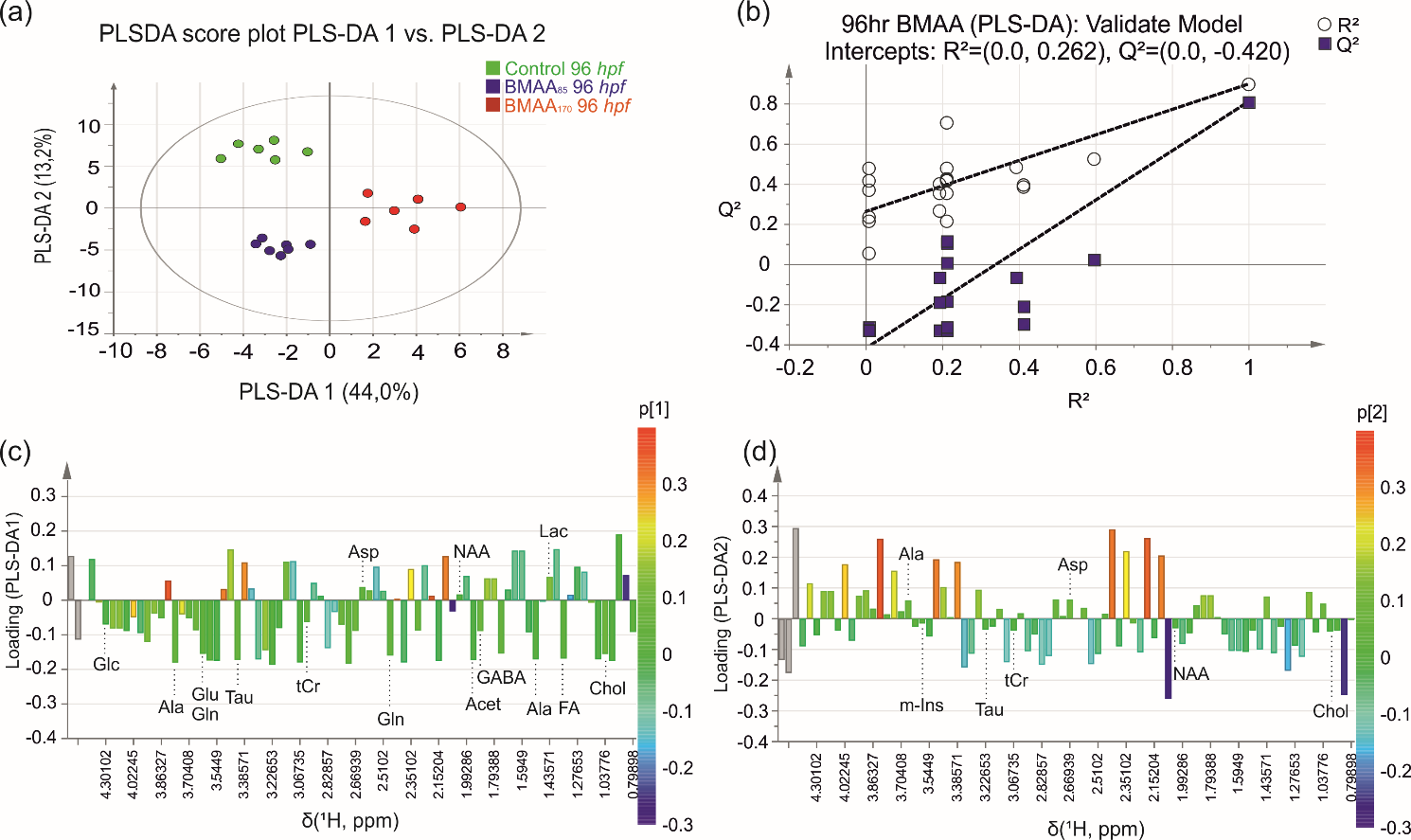


**Supplementary Fig. S12.** (a) PLS-DA scores plots for control 96 hr, BMAA_85_ treated and BMAA_170_ treated embryo. Spectra derived from the same brain tissue have the same color. Total 57.2% variables are used to make this score plot. (b) Model validation plot with R^2^ positive intercepts at (0.0, 0.262) and Q^2^ negative intercepts at (0.0, -0.420). (c) Loading plots of PLS-DA1 and (d) Loading plot of PLS-DA2 for all buckets containing assigned peaks.

**PLS-DA analysis 96hr embryo:** The PLS-DA scores plot of the of first two principle components of the spectra from 96 hr embryo explaining 57.2% of the total variance is shown in SIFig. 12(a) for the three group of samples. A clear clustering could be observed in the score plot of PLS-DA1 vs. PLS-DA2. The control 96hr and BMAA_85_ 96 hr samples were separated by PLS-DA2 scores and control 96 hr and BMAA_170_ 96 hr samples were separated by their PLS-DA1 scores. BMAA_85_ and BMAA_170_ 96hr were also separated by PLS-DA1 scores. Control 96hr clusters towards the negative PLS-DA1 scale and BMAA_170_ 24 hr clusters towards positive PLA-DA1 scale. Model validation plot with R^2^ positive intercepts at (0.0, 0.262) and Q^2^ negative intercepts at (0.0, -0.420) is shown in SIFig. 12(b). In order to determine the variables, i.e. metabolites assigned to the corresponding buckets that are mainly responsible for the separation of three groups, the load values or weights of the PLS-DA1 and PLS-DA2, given in SIFig. 12(c) and (d), were analyzed. Signals coming from Cr, Gln, GABA, Glc, Ala, Glu, Acet, Tau, FA and Chol has negative score in the PLS-DA1 loading plot within 95% significance interval (*P*<0.05) and signal coming from Asp, NAA and Lac has positive score in the in the PLS-DA1 loading plot within 95% significance interval (*P*<0.05). Thus PLS-DA1 loading separates the control from BMAA_170_ treated samples along with BMAA_85_ and BMAA_170_ treated samples and the metabolites responsible for it is also showed the changes upon detection and quantification in the NMR experiments. Loading from PLS-DA2 also shows positive scores for Ala and Asp and negative score for m-Ins, Tau, tCr, NAA and Chol within 95% confidence level (*P*<0.05), thus making it responsible for separation of clusters for control 96hr and BMAA_85_ treated samples with BMAA_170_ treated samples.


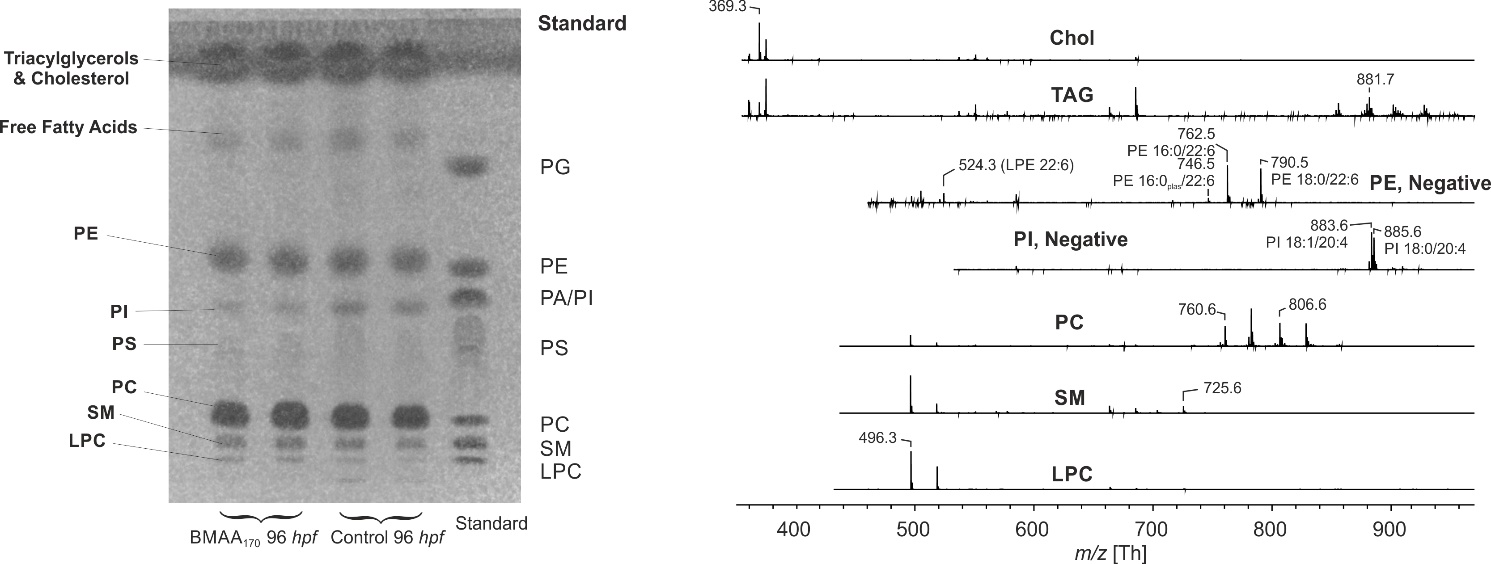


**Supplementary Fig. S13.** (A) Image of a HPTLC plate of a reference PL mixture (1.69 mg each) and zebrafish embryo lipid extract. (B) Positive and negative ion MALDI-TOF mass spectra of the individual PL fractions of the BMAA_170_ treated 96 hpf zebrafish embryo lipid extract recorded directly after HPTLC separation. Even rather "pale" spots (e.g., the PI spot in A) gave high-quality MALDI mass spectra. All peaks are marked according to their m/z ratio. For detailed peak assignments, refer to Table 3. Abbreviations: Gal-Cer, Galacto-cerebrosides; LPE, lysophosphatidylethanolamine; PC, phosphatidylcholine; PE, phosphatidylethanolamine; PI, phosphatidylinositol; SM, sphingomyelin.


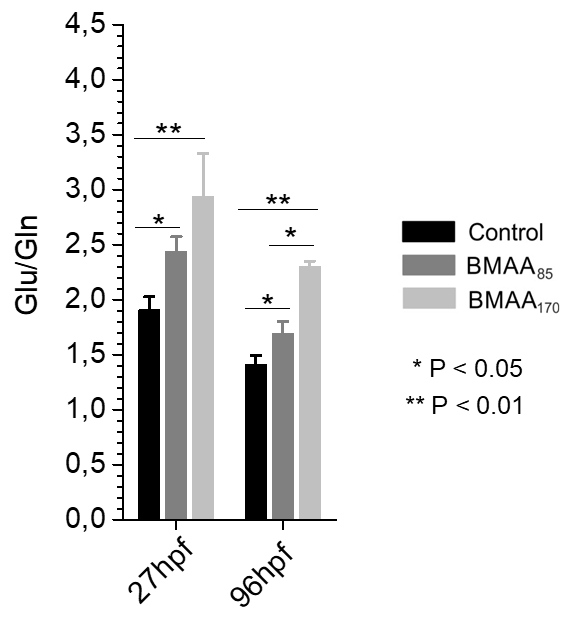


**Supplementary Fig. S14.** Glu-Gln ratio were calculated from the ^1^H HRMAS NMR spectra for both 27 hpf and 96 hpf embryo. The Glu-Gln ratio is increased with BMAA treatment. This hints towards the increased glutaminolysis.


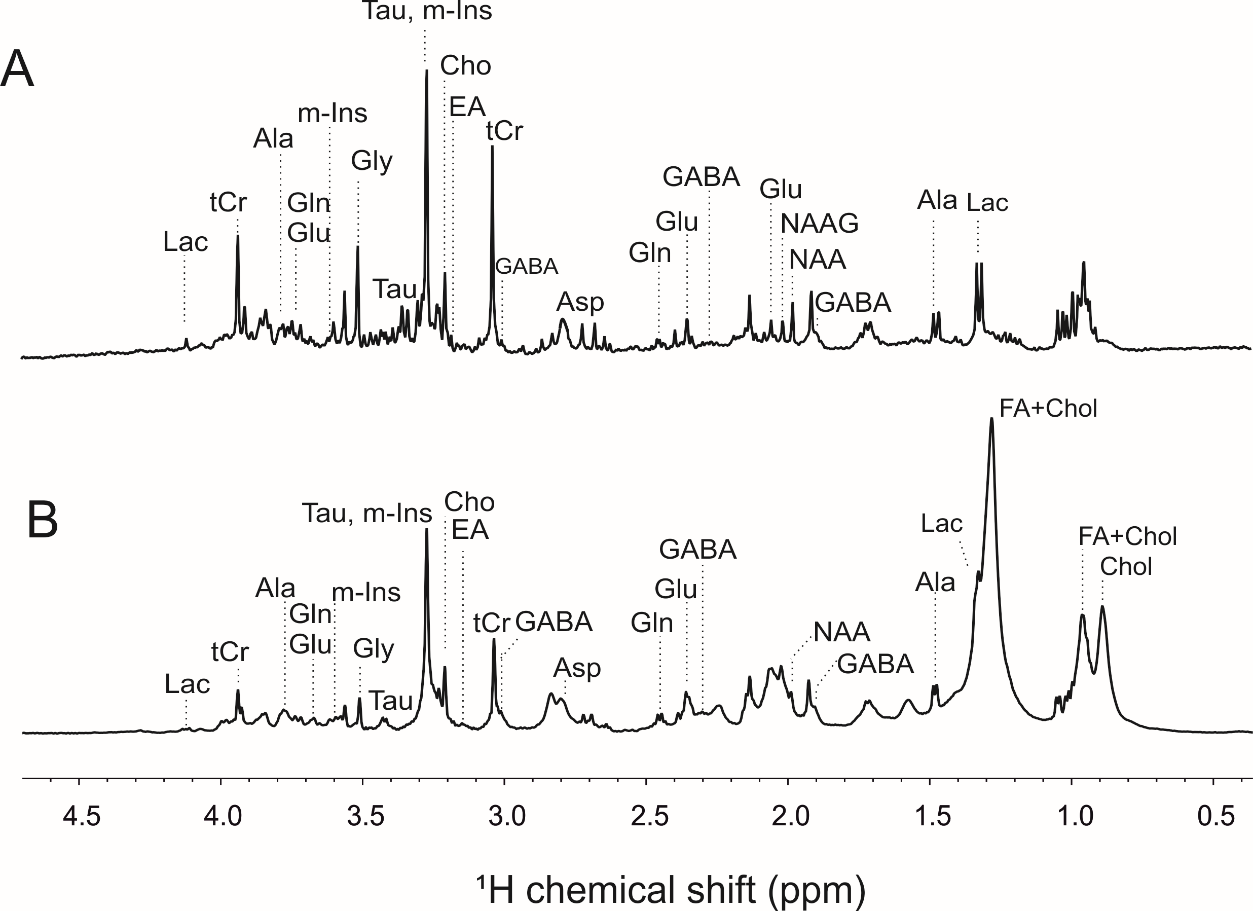


**Supplementary Fig. S15.** Comparison of **(A)** liquid state ^1^H NMR spectra of the metabolic layer extracted from 96 hpf control embryos and (B) HRMAS NMR spectra from intact 96 hpf embryo. The same pattern of chemical shifts are observed in both the spectra confirming the assignments of various metabolites in HRMAS spectra from intact embryos.

**Supplementary Table S1.** ^1^H chemical shifts of metabolites in the zebrafish embryo obtained from 1D and/or 2D NMR spectra at 14.1T.

ψThis work

| **Metabolite** | **Group** | **^1^H chemical shift (ppm)**^ψ^ | **1H chemical shift (ppm) (HMDB)**^φ^ | **Multiplicity** | **Connectivity** |
| --- | --- | --- | --- | --- | --- |
| Ala | ^2^CH_2_ (H^2^)  ^3^CH_3_ (H^3^) | 3.78  1.48 | 3.76  1.46 | q  d | H^2^-H^3^ |
| Arg | ^2^CH (H^2^)  ^3^CH_2_ (H^3^) | 3.76  1.90 | 3.76  1.90 | t  m | H^2^-H^3^ |
| Asp | ^2^CH (H^2^)  ^3^CH_2_ (H^3^)  (H^3’^) | 3.89  2.80  2.66 | 3.89  2.80  2.66 | dd  dd  dd | H^2^-H^3’^  H^3^-H^3’^ |
| Cho | N(CH_3_)_3_  ^1^CH_2_ (H^1^)  ^2^CH_2_ (H^2^) | 3.21  4.07  3.51 | 3.19  4.06  3.51 | s  m  m | -  H^1^-H^2^ |
| Chol | H with C 1, 16,17, 20,22,23  C19  C21  C26  C27 | 1.30  1.00  0.91  0.87  0.85 | 1.3  1.00  0.91  0.87  0.85 | m  s  d  d  d | H^20^-H^21^ |
| tCr | N(CH_3_)  ^2^CH_2_ (H^2^) | 3.03  3.94 | 3.02  3.92 | s  s | - |
| FA | FA (CH_2_)  FA (CH_2_)^a^  FA (terminal CH_3_)^b^ | 1.28  2.05  0.96 | 1.31  2.04  0.98 | s  m  t | -  H^a^-H^b^ |
| Glc | ^1^CH (H^1^)  ^2^CH (H^2^)  ^3^CH (H^3^) & ^5^CH (H^5^)  ^4^CH (H^4^)  ^6^CH (H^6^) | 4.63  3.23  3.46  3.38  3.88 | 4.63  3.23  3.46  3.40  3.88 | d  dd  m  m  dd | H^1^-H^2^  H^2^-H^3^  H^3^-H^4^  H^4^-H^5^  H^5^-H^6^ |
| GABA | ^2^CH_2_ (H^2^)  ^3^CH_2_ (H^3^)  ^4^CH_2_ (H^4^) | 3.00  1.90  2.30 | 3.00  1.89  2.28 | m  m  t | H^2^-H^3^  H^3^-H^4^ |
| Gln | ^2^CH (H^2^)  ^3^CH_2_ (H^3^)  ^4^CH_2_ (H^4^) | 3.75  2.12  2.45 | 3.77  2.13  2.45 | t  m  m | H^2^-H^3^  H^3^-H^4^ |
| Glu | ^2^CH (H^2^)  ^3^CH_2_ (H^3^)  (H^3'^)  ^4^CH_2_ (H^4^) | 3.76  2.12  2.05  2.36 | 3.75  2.12  2.04  2.34 | dd  m  m  m | H^2^-H^3^  H^3'^-H^4^  H^3^-H^4^ |
| Gly | ^2^CH_2_ (H^2^) | 3.52 | 3.54 | s |  |
| GSH (*Glutamate moiety)* | ^3^CH_2_ (H^3^)  ^4^CH_2_ (H^4^) | 2.15  2.54 | 2.15  2.54 | m  m | H^3^-H^4^ |
| PC | ^1^CH_2_ (H^1^)  ^2^CH_2_ (H^2^) | 4.18  3.62 | 4.16  3.60 | m  m | H^1^-H^2^ |
| Ile | ^3^CH (H^3^)  ^4^CH_2_ (H^4^)  ^5^CH_3_ (H^5^)  ^6^CH_3_ (H^6^) | 1.97  1.46  0.92  0.99 | 1.97  1.46  0.92  0.99 | m  m  t  d | H^3^-H^6^  H^4^-H^5^ |
| Lac | ^2^CH (H^2^)  ^3^CH_3_ (H^3^) | 4.13  1.33 | 4.10  1.32 | q  d | H^2^-H^3^ |
| Leu | ^4^CH_2_ (H^4^)  ^5^CH_3_ (H^5^) | 1.70  0.95 | 1.70  0.95 | m  t | H^4^-H^5^ |
| Lys | ^3^CH_2_ (H^3^)  ^4^CH_2_ (H^4^)  ^5^CH_2_ (H^5^)  ^6^CH_2_ (H^6^) | 1.89  1.46  1.71  3.02 | 1.89  1.46  1.71  3.02 | m  m  m  t | H^3^-H^4^  H^5^-H^6^ |
| m-Ins | ^1^CH (H^1^)  ^3^CH (H^3^)  ^4^CH (H^4^)  ^6^CH (H^6^) | 3.56  3.56  3.62  3.62 | 3.52  3.52  3.61  3.61 | dd  dd  t  t | H^3^-H^4^ |
| NAA *Acetyl moiety*  *Aspartate moiety* | ^2^CH_3_ (H^2^)  ^3^CH_2_ (H^3^)  (H^3'^) | 1.99  2.69  2.49 | 2.03  2.70  2.51 | s  dd  dd | H^3^-H^3'^ |
| EA | ^1^CH_2_ (H^1^)  ^2^CH_2_ (H^2^) | 3.14  3.83 | 3.13  3.81 | d  d | H^1^-H^2^ |
| Tau | ^1^CH_2_ (H^1^)  ^2^CH_2_ (H^2^) | 3.42  3.25 | 3.42  3.25 | t  t | H^1^-H^2^ |
| Thr | ^3^CH (H^3^)  ^4^CH_3_ (H^4^) | 4.24  1.32 | 4.24  1.32 | m  d | H^3^-H^4^ |
| Trp | ^α^CH (H^α^)  ^β^CH_2_ (H^β^) | 4.05  3.28 | 4.05  3.29 | dd  dd | H^α^-H^β^ |
| Tyr | ^α^CH (H^α^)  ^β^CH_2_ (H^β^) | 3.93  3.19 | 3.92  3.17 | dd  dd | H^α^-H^β^ |
| Val | ^3^CH (H^3^)  ^4^CH_3_ (H^4^)  (H^4’'^) | 2.26  1.03  0.98 | 2.26  1.03  0.98 | m  d  d | H^3^-H^4^  H^3^-H^4'^ |

φ From HMDB database ([http://www.hmdb.ca](http://www.hmdb.ca/))

**Supplementary Table S2.** Statistical significance on metabolic concentrations upon BMAA treatment for both 24hr and 96hr embryo (^*^ p < 0.05; ^**^ p < 0.01; ^#^ p < 0.001; ^##^ p < 0.0001):

|  | 27hr embryo | | | 96hr embryo | | |
| --- | --- | --- | --- | --- | --- | --- |
| Metabolites | Control vs. BMAA_85_ | Control vs. BMAA_170_ | BMAA_85_ vs. BMAA_170_ | Control vs. BMAA_85_ | Control vs. BMAA_170_ | BMAA_85_ vs. BMAA_170_ |
| NAA | - | - | - | ^#^ F=23.14  p=0.0005 | ^#^ F=26.01  p=0.0004 | - |
| Asp | - | * F=6.92,  p=0.038 | - | ^#^F=27.77  p=0.0004 | ^#^ F=29.24  p=0.0004 | - |
| Glu | - | - | - | * F=8.63  p=0.0164 | ^#^ F=23.22  p=0.0005 | * F=7.56  p=0.0312 |
| Gln | - | * F=7.88,  p=0.0275 | - | ^#^ F=22.66  p=0.0005 | ^#^ F=23.44  p=0.0005 | * F=6.95  p=0.0382 |
| GABA | - | * F=9.51,  p=0.0111 | - | ^#^ F=21.19  p=0.0006 | ^#^ F=23.33  p=0.0005 | - |
| Tau | - | * F= 8.31,  p=0.0202 | - | ^#^ F=29.74  p=0.0004 | ^#^ F=30.37  p=0.0004 | - |
| Chol | ** F= 14.34  p=0.0023 | * F=7.69  p=0.0293 | - | ^##^ F=39.80  p=3.8964x10^-5^ | ^##^ F=50.96  p=1.1823x10^-5^ |  |
| FA | - | ^#^ F=54.30  p=0.0002 | - | ^##^ F=92.72  p=1.4890x10^-7^ | ^##^ F=50.99  p=1.1782x10^-5^ | - |
| Lac | - | * F=9.01  p=0.0151 | - | * F=8.29  p=0.0226 | ^#^ F=26.28  p=0.0004 | * F=8.88  p=0.0157 |
| Ala | **F=15.67  p=0.0020 | ^#^ F=22.17  p=0.0005 | * F=7.99  p=0.0260 | * F=6.78  p=0.0411 | ^#^ F=30.77  p=0.0004 | * F=7.79  p=0.0284 |
| m-Ins | - | * F= 7.15,  p=0.0342 | - | * F=8.17  p=0.0245 | ** F= 16.16,  p=0.0019 | - |
| Gly | ** F=17.58  p=0.0015 | ** F=14.99  p=0.0021 | - | ^#^ F=27.22  p=0.0004 | ^#^ F=29.11  p=0.0004 | - |
| EA | - | - | - | *F=7.10  p=0.0355 | ^#^ F=23.17089  p=0.0005 | *F=6.81  p=0.0401 |
| Glc | - | - | - | ^#^ F=33.96  p=0.0003 | ^##^ F=43.11  p=0.0003 | **F=18.72  p=0.0011 |
| GSH | * F=9.71  p=0.0107 | * F=9.93  p=0.0100 | - | * F=7.42  p=0.032 | * F=8.13  p=0.0251 | - |

**Supplementary Table S3.** Survey of the peaks detected in the positive and negative ion MALDI-TOF mass spectra of the BMAA_170_ treated 96 hpf zebrafish embryo lipid extract and the corresponding assignments^a^. Spectra were recorded subsequent to separation by HPTLC.

| Peak position (*m/z*) | Assignment of molecular mass^b^ |
| --- | --- |
| 369.3 | Chol |
| 496.3 | LPC |
| 524.3 | LPE 18:1 (–H^+^ +2 Na^+^) |
| 725.6 | SM 16:0 (+Na^+^) |
| 746.5 | PE 16:0/18:1 (+Na^+^) |
| 756.6 | PC 16:0/16:0 (+Na^+^) |
| 762.5 | PE 16:0/22:6 (+H^+^) |
| 782.6 | PC 16:0/18:1 (+Na^+^) |
| 790.5 | PE 18:1/20:4 (+Na^+^) |
| 806.6 | PC 16:0/22:6 (+H^+^) |
| 810.6 | PC 18:0/18:1 (+Na^+^) |
| 828.6 | PC 16:0/22:6 (+Na^+^) |
| 883.6 | PI 18:1/20:4 (+H^+^) |
| 885.6 | PI 18:0/20:4 (+Na^+^) |
| 881.7 | TAG |

^a^All PL besides SM and PC contain functional groups showing exchange with the solvents and/or ions of the matrix solution leading to complex peak patterns.

^b^Abbreviations: Chol, cholesterol; LPC, lysophosphatidylcholine; LPE, lysophosphatidylethanolamine; PC, phosphatidylcholine; PE, phosphatidylethanolamine; PI, phosphatidylinositol; SM, sphingomyelin; TAG, triacylglycerois.

**References**

1 van Amerongen, Y. F. *et al.* Zebrafish Brain Lipid Characterization and Quantification by ^1^H Nuclear Magnetic Resonance Spectroscopy and MALDI-TOF Mass Spectrometry. *Zebrafish* **11**, 240-247 (2014).

2 Westerfield N. (2005) The zebrafish book. The guide for the laboratory use of zebrafish (Danio rerio). 4th edition. Eugene: University of Oregon Press.

3 Ali, S., Mil, H. G. J. v. & Richardson, M. K. Large-Scale Assessment of the Zebrafish Embryo as a Possible Predictive Model in Toxicity Testing. P*LOS ONE* 6**,** e21076, doi:10.1371/journal.pone.0021076 (2011).

4 Suhartono, L. *et al.* Metabolic comparison of cryopreserved and normal cells from Tabernaemontana divaricata suspension cultures. P*lant Cell Tiss Organ Cult* 8**3,** 59-66, doi:10.1007/s11240-005-3869-8 (2005).

5 Fuchs, B., Nimptsch, A., S, R. & Schiller, J. Analysis of Brain Lipids by Directly Coupled Matrix-Assisted Laser Desorption Ionization Time-of-Flight Mass Spectrometry and High-Performance Thin-Layer Chromatography. J*ournal of AOAC International* 9**1,** 1227-1236 (2008).

6 Fuchs, B. *et al.* Analysis of stem cell lipids by offline HPTLC-MALDI-TOF MS. A*nalytical and Bioanalytical Chemistry* 3**92,** 849-860, doi:10.1007/s00216-008-2301-8 (2008).

7 White, T., Bursten, S., Federighi, D., Lewis, R.A., Nudelman, E (1998) High resolution separation and quantification of neutral lipid and phospholipid species in mammalian cells and sera by multi-one-dimensional thin-layer chromatography. *Anal Biochem* 258, 109–117.

8 Fuchs, B., Schiller, J., Süß, R., Schürenberg, M. & Suckau, D. A direct and simple method of coupling matrix-assisted laser desorption and ionization time-of-flight mass spectrometry (MALDI-TOF MS) to thin-layer chromatography (TLC) for the analysis of phospholipids from egg yolk. Ana*lytical and Bioanalytical Chemistry 38*9**, 8**27-834, doi:10.1007/s00216-007-1488-4 (2007).

9 Sun, G. et *al. Sh*otgun Metabolomics Approach for the Analysis of Negatively Charged Water-Soluble Cellular Metabolites from Mouse Heart Tissue. Ana*lytical chemistry 79*, **6**629-6640, doi:10.1021/ac070843+ (2007).
